# Deciphering the impact of genetic variation on human polyadenylation using APARENT2

**DOI:** 10.1186/s13059-022-02799-4

**Published:** 2022-11-05

**Authors:** Johannes Linder, Samantha E. Koplik, Anshul Kundaje, Georg Seelig

**Affiliations:** 1 Department of Genetics, Stanford University, Stanford, USA; 2 Department of Bioengineering, University of Washington, Seattle, USA; 3 Department of Computer Science, Stanford University, Stanford, USA; 4 Paul G. Allen School of Computer Science and Engineering, University of Washington, Seattle, USA; 5 Department of Electrical and Computer Engineering, University of Washington, Seattle, USA

**Keywords:** RNA, Polyadenylation, Deep learning, Neural networks, Untranslated region, Variant interpretation, Genomics, Explainable AI

## Abstract

**Background:**

3′-end processing by cleavage and polyadenylation is an important and finely tuned regulatory process during mRNA maturation. Numerous genetic variants are known to cause or contribute to human disorders by disrupting the cis-regulatory code of polyadenylation signals. Yet, due to the complexity of this code, variant interpretation remains challenging.

**Results:**

We introduce a residual neural network model, *APARENT2*, that can infer 3′-cleavage and polyadenylation from DNA sequence more accurately than any previous model. This model generalizes to the case of alternative polyadenylation (APA) for a variable number of polyadenylation signals. We demonstrate APARENT2’s performance on several variant datasets, including functional reporter data and human 3′ aQTLs from GTEx. We apply neural network interpretation methods to gain insights into disrupted or protective higher-order features of polyadenylation. We fine-tune APARENT2 on human tissue-resolved transcriptomic data to elucidate tissue-specific variant effects. By combining APARENT2 with models of mRNA stability, we extend aQTL effect size predictions to the entire 3′ untranslated region. Finally, we perform in silico saturation mutagenesis of all human polyadenylation signals and compare the predicted effects of ${>}43$ million variants against gnomAD. While loss-of-function variants were generally selected against, we also find specific clinical conditions linked to gain-of-function mutations. For example, we detect an association between gain-of-function mutations in the 3′-end and autism spectrum disorder. To experimentally validate APARENT2’s predictions, we assayed clinically relevant variants in multiple cell lines, including microglia-derived cells.

**Conclusions:**

A sequence-to-function model based on deep residual learning enables accurate functional interpretation of genetic variants in polyadenylation signals and, when coupled with large human variation databases, elucidates the link between functional 3′-end mutations and human health.

**Supplementary Information:**

The online version contains supplementary material available at 10.1186/s13059-022-02799-4.

## Background

**Fig. 1:**
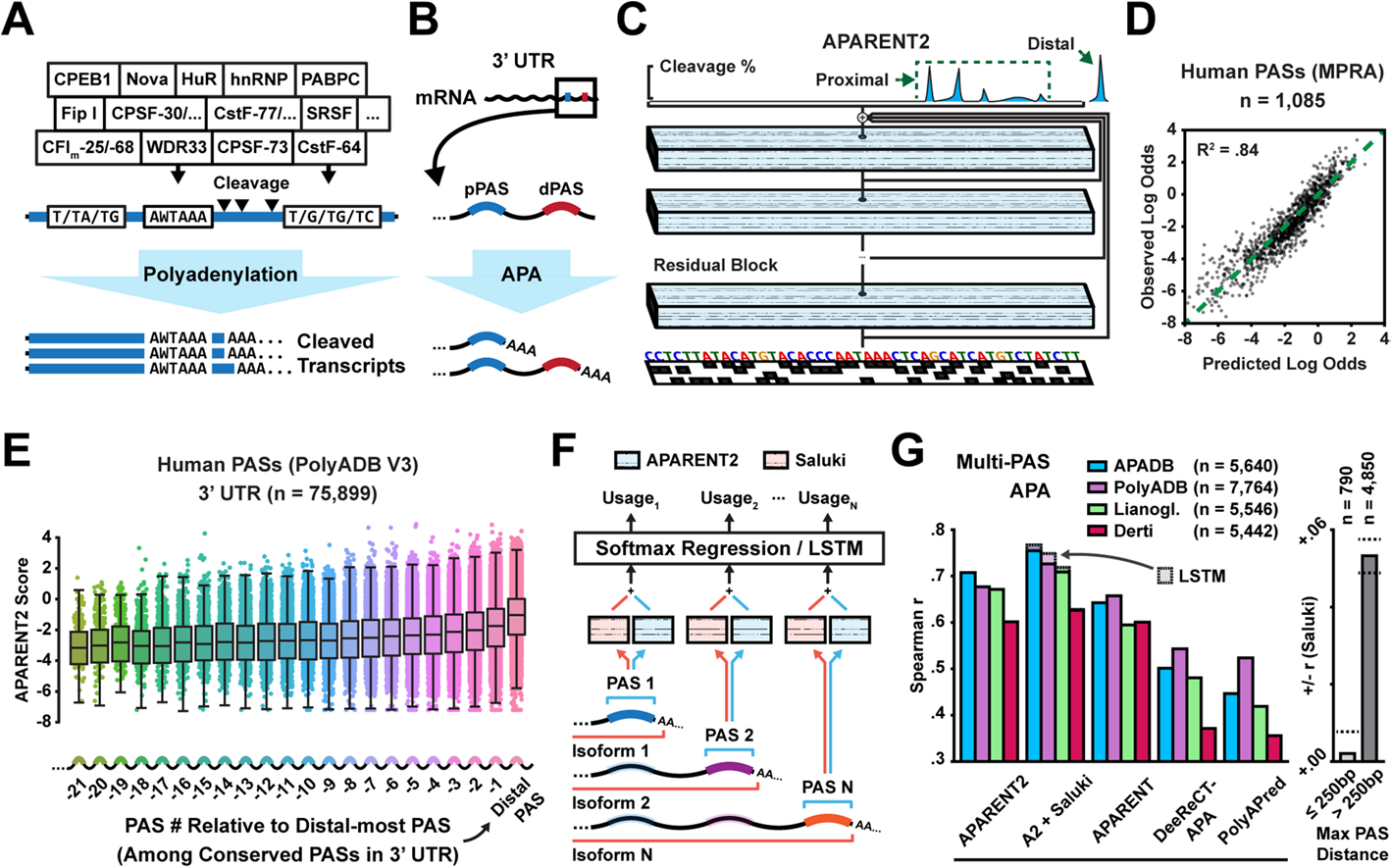
A deep residual neural network for predicting polyadenylation. **A** Core processing elements, auxiliary RBPs, and other determinants influence polyadenylation signal affinity. **B** Illustration of tandem 3′ UTR alternative polyadenylation (APA) in pre-mRNA. **C** Residual neural network architecture. A one-hot coded representation of the PAS is used to predict the 3′ cleavage distribution. **D** Predicted vs measured proximal isoform log odds of native human 3′ UTR PASs measured in an MPRA ($n=1085$). **E** Predicted logit score of all human PASs as a function of PAS # relative to the distal-most PAS. **F** Masked softmax regression (or a LSTM) for predicting multi-PAS isoform proportions given APARENT2 and Saluki scores as input. **G** Left: Comparison of correlation between predicted and measured distal isoform proportions from tissue-pooled native data (20-fold cross-validation). Each model predicts logit scores which are used to fit a multi-PAS regressor. LSTM performance shown as shaded bars. Right: Improvement in Spearman *r* when using Saluki scores in addition to APARENT2 as input; the improvement is shown separately for genes where the maximum distance between any adjacent pair of PASs is ${\le }250\mathrm{bp}$ and ${>}250\mathrm{bp}$ respectively

Almost all human mRNA transcripts undergo cleavage and polyadenylation (pA). The position and efficiency of 3′ cleavage are controlled by a complex cis-regulatory code, the polyadenylation signal (PAS) (Fig. 1A). The PAS consists of a core hexamer, typically AATAAA, and surrounding upstream and downstream sequence elements which together recruit the core processing machinery (CFIm, CstF, CPSF, and hFIP1) [1–4]. A large number of auxiliary factors, including hnRNP F/H/I, SRSF proteins, PABPC1, Ptbp2, HuR, and Nova, further modulate pA strength by binding to sequence motifs in the PAS [5–10]. Adding an extra layer of complexity, the exact variants of these motifs, their relative positioning, and interactions with structural motifs such as stem loops determine their cooperative, or antagonistic, effects [11]. Moreover, more than 70% of human genes contain multiple PASs (alternative polyadenylation, or APA), resulting in RNA isoforms with distinct 3′ ends (Fig. 1B) [12–14]. The most common form of APA is the occurrence of two or more competing PASs in the 3′ untranslated region (3′ UTR) [1]. While all isoforms code for the same protein, their characteristics such as RNA stability or translation efficiency may vary considerably, as miRNA binding sites and other regulatory elements could have been removed from the shorter isoforms [15]. Less commonly, polyadenylation can also occur within introns, resulting in truncated protein isoforms.

Assessing the impact of genetic variation on pA is important in both research and clinical settings, as several mutations that disrupt APA isoform abundances have been implicated in disease [16–18]. Even single PASs in 3′ UTRs without competing signals may have finely tuned functions, as weak mutations in such PASs can affect mRNA abundance [19]. While genome-wide association studies (GWAS) and mapping of APA QTLs (3′ aQTLs) are powerful tools for finding statistical links between variants and phenotype [20, 21], they require a relatively large sample size and are less useful for rare or de novo variants [22, 23]. In a complementary approach, deep learning models that predict the *functional* impact of variants from sequence have been successful at classifying disruptive mutations, regardless of population frequency [24–31]. Such sequence-predictive models have even been developed for pA [32–35]. In particular, we previously trained a convolutional neural network (CNN) called APARENT for APA prediction [36].

Inspired by the recent success of deep residual networks applied to splicing and transcription factor binding prediction [27, 37], we here introduce *APARENT2*, a sequence-based residual neural network for 3′ cleavage prediction at base-resolution. We systematically compare the performance of APARENT2 to other models at the task of predicting disruptive variants, using functional MPRA data of 12,350 single nucleotide variants (SNVs) from ClinVar and HGMD or from saturation mutagenesis of clinically relevant genes [36, 38–40] as well as scanning mutagenesis data from an assay of more than 12,000 PASs [35]. We further compare the models on 366 high-confidence human 3′ aQTLs in 44 tissues from GTEx V7 [20], 1223 aQTLs from the newer GTEx V8 atlas [41], and 58 aQTLs measured among 52 HapMap Yoruba human lymphoblastoid cell lines [21]. In all tests, APARENT2 outperforms all state-of-the-art APA models. By combining APARENT2 with auxiliary tissue-specific models that we learn from native transcriptomic data in the testis, ovary, B-cell lymphocytes, and brain [42], we provide residual variant predictions that boost performance on GTEx 3′ aQTLs. Finally, using a linear model to combine the predictions of APARENT2 and a separate model of mRNA stability [43], we further refine our 3′ aQTL scores by accounting for differential isoform stability. This generalized model allows for scoring any variant in the entire 3′ UTR.

In silico interpretation methods have been applied extensively to assess the impact of genetic variants on the underlying cis-regulatory code [30, 37, 44–48]. Here, we use a mask-based interpretation method for neural networks—Scrambling—to elucidate higher-order features responsible for the predicted variant effects [49]. Specifically, we extend Scramblers to find the minimal set of features which explain the functional differences between a variant and wildtype sequence. With this approach, we discover super-additive interactions such as those between the CFIm-binding motif TGTA and AT-rich elements, or motifs that are differentially more active in the brain and testis. We also find that some human PASs contain protective core hexamers (CSEs) that can initiate polyadenylation when the main CSE is disrupted. To understand the evolutionary constraints of polyadenylation in humans, we cross-reference the predicted effects of all potential 44 million polyadenylation SNVs against the 2.8 million PAS variants observed in gnomAD [50]. We find that loss-of-function variants occur ${\sim } 2.5$-fold less frequently in common variants (AF ${>} 10\%$) compared to singletons. However, when applying APARENT2 to a cohort study of autism spectrum disorder (ASD), we found a ${\sim } 3$-fold enrichment of gain-of-function PAS mutations in cases (Fisher’s exact *p* = $2.2{\times }10^{-4}$) [51]. To experimentally validate our predictions, we measured the impact of 94 clinically relevant variants (including variants from ASD cases and associated controls) in an MPRA in HEK293T, SK-N-SH, and HMC3 cell lines.

## Results

### A residual neural network for predicting 3′ cleavage and polyadenylation

Given recent advances in deep learning, we first asked whether an updated neural network architecture could improve on the performance of current state-of-the-art predictors such as APARENT. To this end, we trained a deep residual network on a re-processed version of the APA MPRA of Bogard et al. [36]. These data contain ${>}3.3$ million APA reporters with randomized proximal PAS sequence measured within 12 diverse 3′ UTR contexts. Briefly, the MPRA data was re-processed to map 3′ cleavage reads at base-pair resolution for some missing UTR contexts (see Section 5 for details). The network, which is illustrated in Fig. 1C and is referred to as APARENT2, is architecturally similar to SpliceAI [27] and BPNet [37]. Through a sequence of 28 residual blocks [52], each block consisting of two layers of dilated convolutions and a skip connection (Additional file 1: Fig. S1A-B), the network transforms a one-hot coded representation of the input PAS (205 nt) into a predicted 3′ cleavage distribution. The last (206th) output of the network predicts the total isoform proportion of a far-away competing distal PAS (which in the training MPRA is non-random). For baseline comparisons, we also retrained a model with the original APARENT architecture on the re-processed version of the same MPRA (referred to as ConvNet below). To evaluate performance, we tested each network’s ability to infer total proximal isoform abundance on a held-out set of 1085 native human PASs (also measured in the MPRA [36]) (Fig. 1D). APARENT2 had significantly better correlation ($R^2 = 0.84$) compared to the ConvNet baseline ($R^2 = 0.77$; Additional file 1: Fig. S1C). APARENT2 also had better correlation on held-out test data from the random MPRA (Additional file 1: Fig. S1D).

Although APARENT2 was trained in the context of tandem APA, we note that the network effectively learns to score PASs relative to a fixed reference and we can thus interpret this score as an absolute measurement of PAS strength. Using APARENT2 as a PAS scoring function, we applied it to all human PASs in PolyADB V3 [53, 54]. In agreement with earlier analyses suggesting that distal signals are functionally more conserved [55], we found a near-perfect monotonically decreasing trend in predicted cis-regulatory strength as a function of PAS rank relative to the distal-most PAS of each gene (Fig. 1E). The median strength of the proximal-most PAS was reduced ${\sim }6$-fold compared to the distal-most PAS (Additional file 1: Fig. S1E). We also successfully recapitulated binding motifs for several known pA mediators, including CFIm, CstF, HNRNPH2, and HuR by applying a motif discovery method, TF-MoDISco [56], to the APARENT2 predictions of 20,000 PAS sequences from PolyADB (Additional file 1: Fig. S1F-G).

In the context of a multi-PAS gene, isoform abundance of a given PAS is determined not only by its intrinsic strength but also by the relative strength and distance of competing signals. Additionally, isoform abundances may be affected by the differential mRNA stability of the resulting 3′ UTR isoforms. To predict isoform proportions for genes with arbitrary numbers of PASs, we thus used native 3′-sequencing data to fit a multi-PAS regression model using the APARENT2 scores, the PAS distances, and a PCA reduction of the hidden-layer embedding of each isoform predicted by the Saluki model [43], as inputs (Fig. 1F; Spearman *r* ranged between 0.63 and 0.76 depending on data source when comparing measured to predicted distal isoform proportions with 20-fold cross-validation) [13, 42, 54, 57]. Using the hidden-layer embeddings instead of the final Saluki half-life predictions consistently improved performance. When comparing to other APA models, including PolyApredictor [35], DeepPASTA [33], and DeeReCT-APA [34], APARENT2 was the most accurate at the task of multi-PAS prediction (Fig. 1G) and pairwise PAS prediction (Additional file 1: Fig. S1H-I). Switching the softmax regression layer of the multi-PAS model for a recurrent network (a LSTM [58]) resulted in only marginal performance gains (*r* increased by 0.0 to 0.025; Fig. 1G).

While the overall improvement to predictive performance increased only modestly when including the Saluki half-life scores as input (Spearman *r* increased by 0.047 on the APADB data), we noted that the improvement increased monotonically with larger differences between isoform lengths (Fig. 1G, right; Additional file 1: Fig. S1J). For genes with large PAS distances (${>}250$bp), a larger predicted difference in isoform stability was associated with larger improvement to predictive performance, while for genes with short isoforms (${\le }250$bp), there was no improvement even for highly differentially stable transcripts (Additional file 1: Fig. S1K). Taken together, these results suggest that APARENT2 can score cis-regulatory stability elements near the PAS, but that a more general stability model such as Saluki is beneficial for 3′ UTRs with long isoforms.

### Predicting the impact of variants on polyadenylation signal processing efficiency

**Fig. 2:**
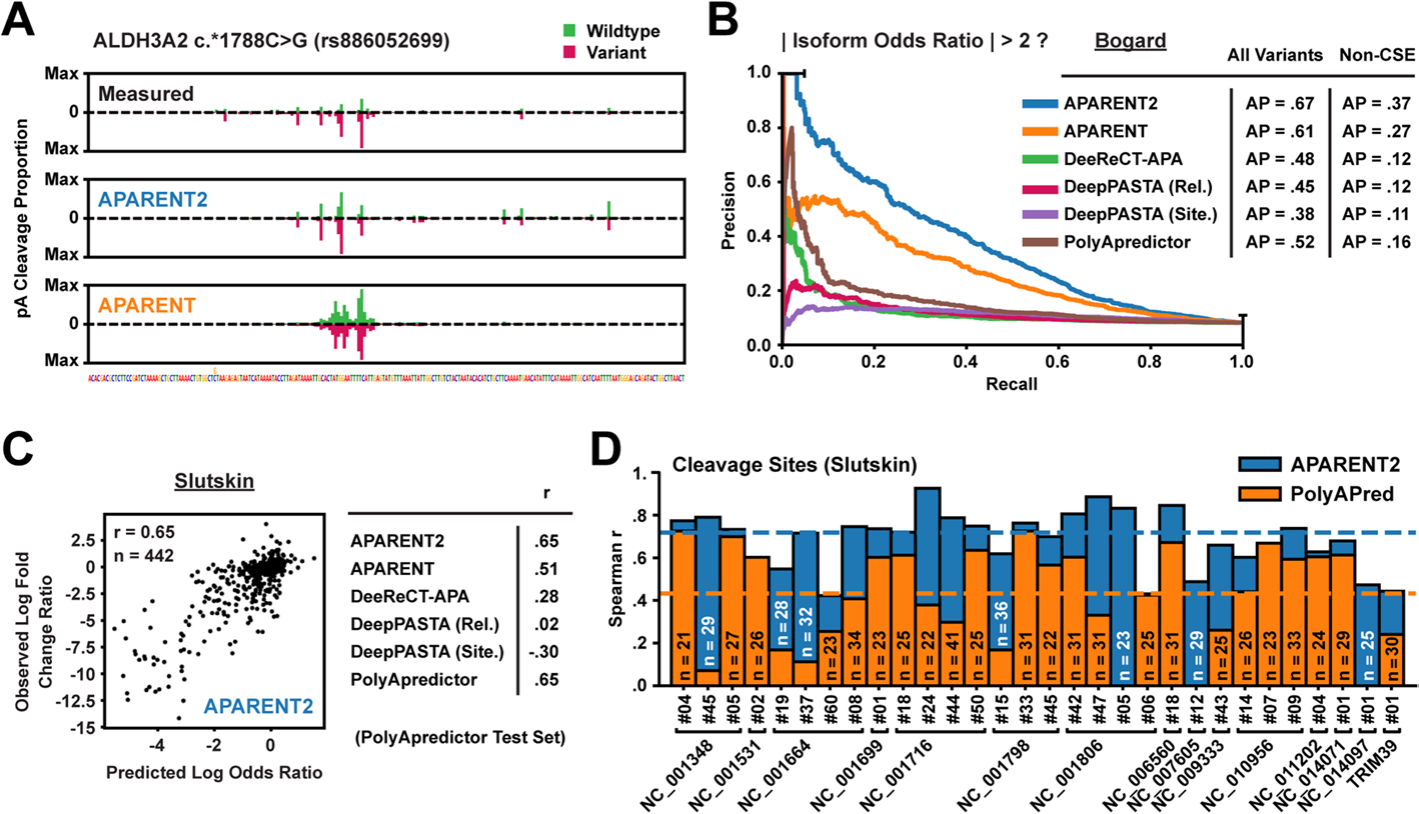
Prediction of functionally screened polyadenylation variants. **A** Variant of uncertain significance from ClinVar (rs886052699) measured in an MPRA [36]. Shown are the measured and predicted 3′ cleavage distributions across the PAS. Green: wildtype cleavage, magenta: variant cleavage. **B** Comparison of precision-recall curves when tasking each APA model with classifying disruptive APA variants ($|\text {fold change}| > 2$) from the MPRA of Bogard et al. [36] (*n* = 12,350). The curves are shown for non-CSE variants only. **C** Comparison of predicted vs measured RNA/DNA log fold change ratios on the data from Slutskin et al. [35] (*n* = 442). **D** Comparison of predicted vs measured RNA/DNA log fold change ratios at individual cleavage sites within a given PAS

We next compared APARENT2 to APARENT, DeepPASTA, DeeReCT-APA, and PolyApredictor at the tasks of classifying disruptive variants and estimating effect sizes (see Section 5 for details on how each model was used). We first analyzed our own variant MPRA [36], consisting of 12,350 SNVs occurring near PASs of disease-implicated 3′ UTRs from ClinVar, HGMD, or ACMG genes [38–40]. Figure 2A shows that the wildtype and variant cleavage distributions predicted by APARENT2 match the measured peaks better than the original APARENT model for an example SNV (rs886052699) in the ALDH3A2 gene. When comparing all models based on their ability to predict isoform fold changes and classify disruptive variants (|fold change| $> 2$), we found that APARENT2 had the highest overall accuracy (Fig. 2B, Additional file 1: Fig. S2A-B; average precision = 0.67; $R^{2} = 0.69$; $n = 12,350$). Importantly, the performance gap of APARENT2 increased when looking only at the more challenging class of variants outside of the CSE.

We further compared the models on a separate 3′ UTR MPRA which measured expression levels as a proxy for polyadenylation processing efficiency [35]. In this assay, a single PAS was inserted in each mini-gene reporter and RNA levels were found to vary over almost an order of magnitude with PAS strength and thus 3′-end processing efficiency. These data allow us to test our ability to infer intrinsic PAS strength independent of the presence of APA. We tested the models on a subset of scanning mutagenesis measurements of several native PASs, including 572 viral PASs. We first compared the models on how well their predicted variant fold changes correlated with measured RNA/DNA fold change ratios (Fig. 2C, Additional file 1: Fig. S2C). Here, APARENT2 and PolyApredictor, a model trained directly on these data, had identical correlations ($\text {Spearman } r = 0.65$, $n = 442$), which was considerably higher than other models. However, when predicting variant fold change ratios at individual cleavage sites, APARENT2 was more accurate (Fig. 2D, Additional file 1: Fig. S2D; median $\text {Spearman } r = 0.72$, total $n=1217$).

### A catalog of higher-order polyadenylation variant interpretations

**Fig. 3:**
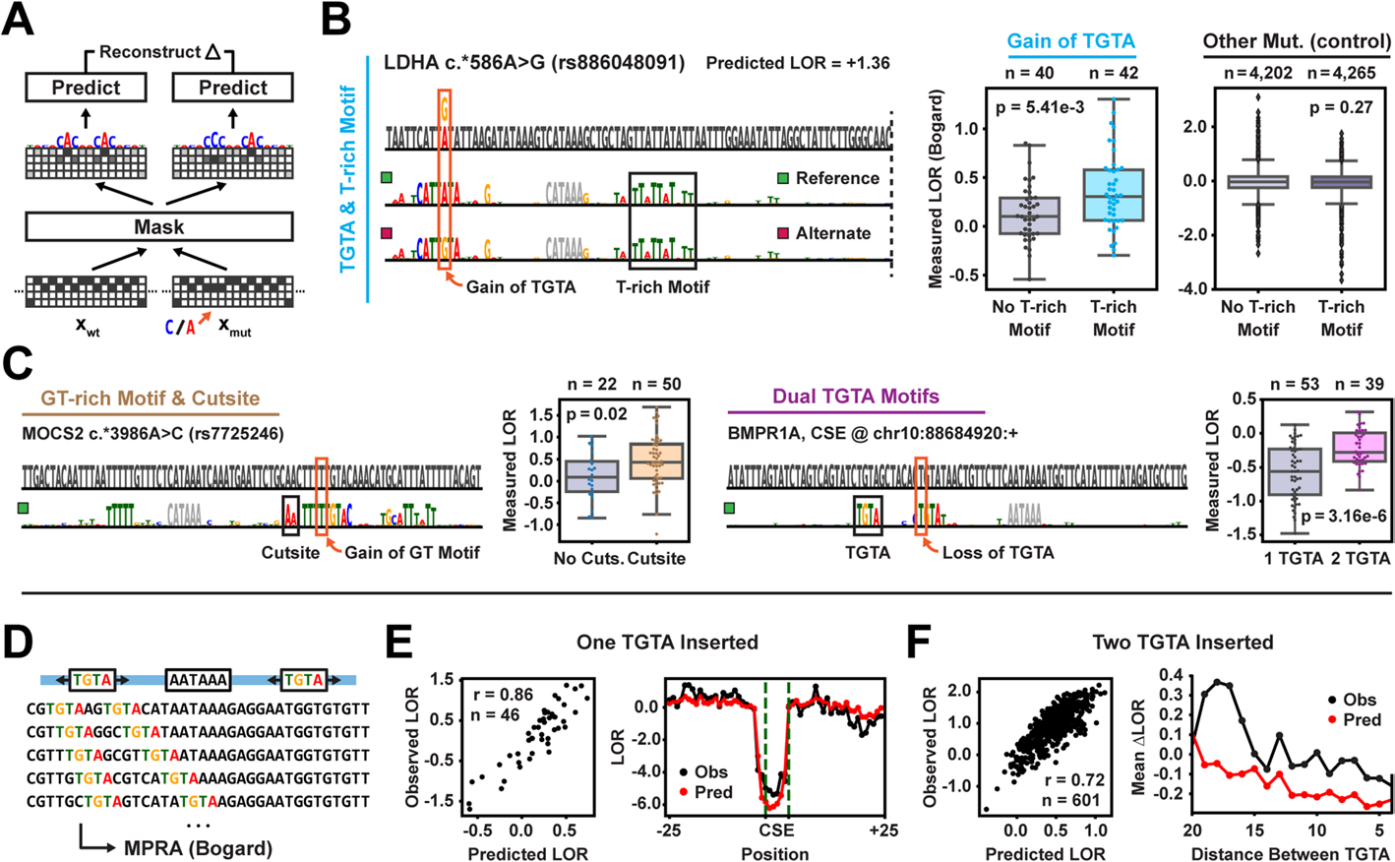
Interpretation of cis-acting polyadenylation variants. **A** Mask-based variant interpretation, reconstructing the relative odds ratio between the wildtype and mutated sequence. **B** Interpretation of a ClinVar SNV in the LDHA PAS (rs886048091). Left boxplot: Measured LORs of TGTA-creating variants from the MPRA of Bogard et al. [36]. Right boxplot: Measured LORs of non-TGTA-creating variants. *p*-values are computed with two-sided *t*-tests. **C** Interpretation of two variants of interest in the MOCS2 PAS and BMPR1A PAS. **D** Individual- and pairwise TGTA motifs were inserted in wildtype PASs and their LORs were measured in an MPRA [36]. **E** Predicted and observed LOR of individual TGTA insertions. **F** Predicted and observed LOR of dual TGTA insertions

Given the increased performance of a more complex neural network architecture (in this case a deep residual network), we wanted to understand the types of higher-order regulatory features learned by APARENT2 that impact variant effect predictions in polyadenylation signals. To this end, we used a neural network attribution method recently developed by our group—Scrambling—to detect contextual features responsible for the observed variant effects [49]. To interpret a mutation, we optimize a discretized mask to highlight a shared set of features (nucleotides) in the wildtype and variant sequences that allows reconstruction of their predicted odds ratio when inserted into neutral backgrounds (Fig. 3A; see Section 5 for details).

In Fig. 3B, we interpret a gain-of-function variant (rs886048091) which creates an upstream CFIm-binding motif (TGTA) and is both predicted and measured to have a log odds ratio (LOR) that is larger than the median fold change observed for other TGTA-creating mutations (predicted LOR = $+1.36$, median LOR = $+0.17$). The mask-based interpretation elucidates a cooperative interaction with a downstream T-rich motif, which reconstructs the prediction. We find additional support in the MPRA data of Bogard et al. [36], as T-rich elements in the DSE are associated with higher-magnitude TGTA-creating mutations ($p = 5.41 \times 10^{-3}$; Fig. 3B, right), while T-rich elements are not associated with larger effect sizes for other mutations ($p = 0.27$). Additionally, rs886048091 stabilizes the RNA secondary structure of the PAS [59] (Additional file 1: Fig. S3A) and the interpretation highlights altered base-pairing positions near the mutation.

We similarly interpret two other SNVs with markedly high or low effect sizes which create putative CstF-binding and CFIm-binding motifs (Fig. 3C). Our interpretations elucidate cooperative and competitive interactions with a nearby 3′ cleavage site and a nearby redundant TGTA motif respectively. The MPRA measurements also indicate significant super- and sub-additive variant effect sizes in the presence of the proposed interacting motifs ($p = 0.02$ and $p = 3.16 \times 10^{-6}$) [36]. In Additional file 1: Fig. S3B-D, we describe additional higher-order features in the context of SNVs. For example, we find that well-positioned T-rich elements are crucial determinants of de novo cleavage when mutations create competing CSE hexamers.

The mask in Fig. 3C (right) highlights an overall sub-additive (competitive) relationship between CFIm-binding motifs. To study this interaction in detail, we re-analyzed a subset of our previously published MPRA data [36] where either single or dual TGTA motifs were inserted at all possible positions of three randomly selected wildtype sequences and their impact on pA efficiency was measured with respect to the wildype activity (Fig. 3D). APARENT2 could accurately predict the LOR of both individual TGTA insertions (Fig. 3E; Additional file 1: Fig. S3E; Spearman $r = 0.86$) and dual insertions (Fig. 3F; Additional file 1: Fig. S3F; Spearman $r = 0.72$). APARENT2 predicts an increasingly sub-additive trend with decreasing motif distance, which is consistent with the MPRA measurements.

### Silent hexamer mutations are protected by functional redundancy

**Fig. 4:**
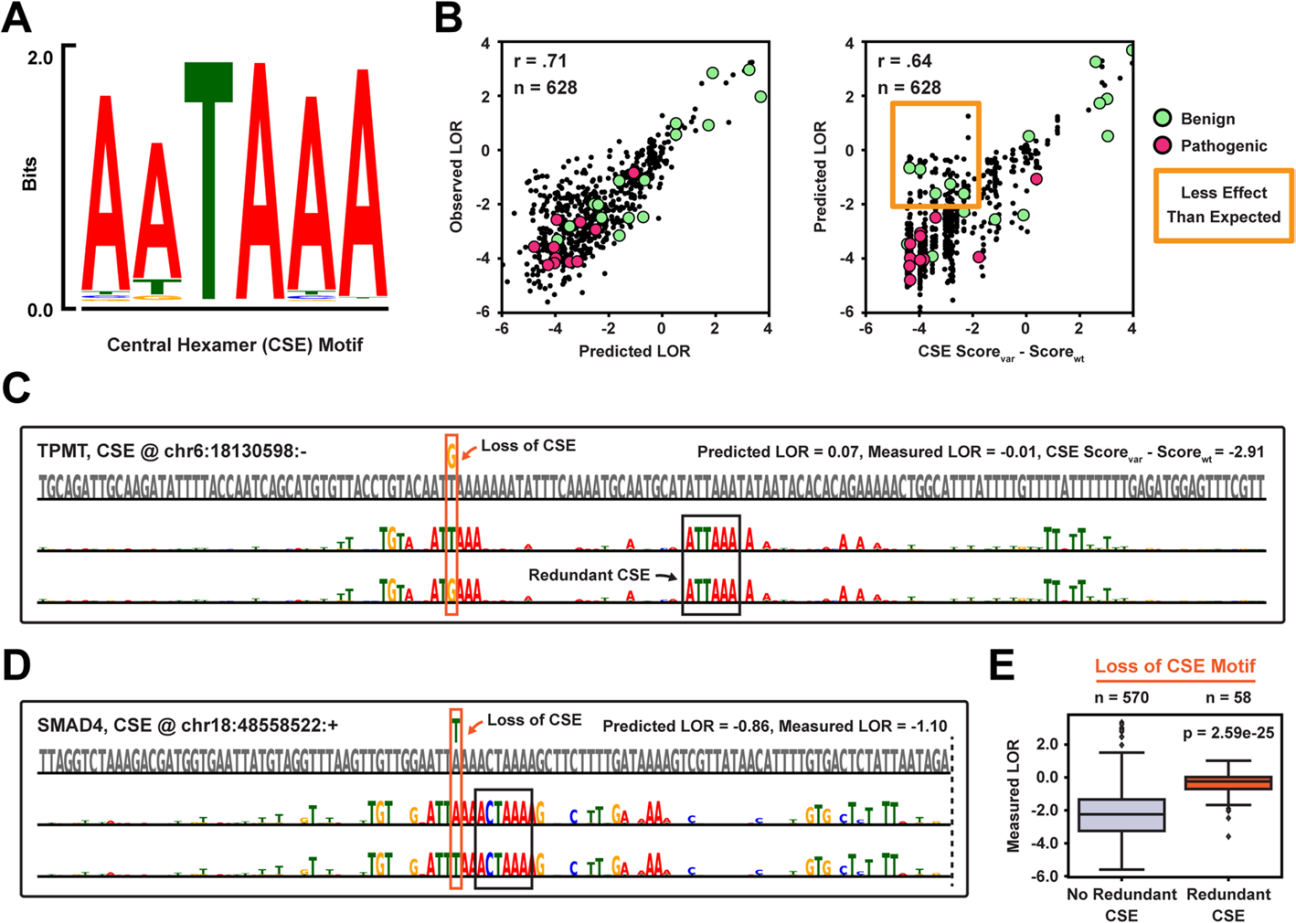
Redundancy of functional hexamer motifs in human polyadenylation signals. **A** Position weight matrix (PWM) of the CSE motif, as measured in the MPRA of Bogard et al. [36]. **B** Predicted vs measured log odds ratio of CSE mutations from the MPRA (*n* = 628). Right: Log odds ratio predicted by APARENT2 vs the effect sizes predicted by a linear hexamer model trained on the same data. **C** Interpretation of a functionally silent CSE mutation in the TPMT gene. **D** Interpretation of a variant with dampened effect size in the SMAD4 gene. **E** Boxplot showing measured LORs of all assayed CSE mutations [36]. *p*-values are computed with two-sided *t*-tests

The core cis-regulatory polyadenylation element in humans is the CSE hexamer motif, which in its canonical form is either AATAAA or ATTAAA but weaker nucleotide variants exist (Fig. 4A) [1]. Reporter experiments measuring polyadenylation efficiency have recently shown that clinically benign CSE mutations often have lower functional effect sizes than expected [60]. To investigate this phenomenon at a larger scale, we collected all measured CSE mutations from the MPRA of Bogard et al. [36] (*n* = 628) and compared APARENT2’s variant effect predictions to the measurements (Fig. 4B, left). APARENT2 can regress the effect sizes accurately (Spearman *r* = 0.71) and the predictions generally separate the benign from pathogenic labels in ClinVar.

To identify CSE variants with predicted effect sizes that are lower than expected, we compared APARENT2’s predictions to a linear CSE hexamer regression model trained on the same data (Fig. 4B, right). While the two models generally agree (Spearman *r* = 0.64), we find multiple mutations with log odds ratios $< -2$ as predicted by APARENT2 but with log odds ratios $> -2$ as predicted by the hexamer model. All variants that occur in ClinVar within this group are labeled benign. Using our mask-based interpretation method, we dissected the origin of this discrepancy. First, we find a group of completely silent mutations and these PASs all contain redundant CSE hexamers (Fig. 4C, Additional file 1: Fig. S4A). Importantly, the interpretations show that besides the extra CSE motifs, it is crucial that auxiliary elements (e.g., CFIm-binding TGTA motifs or downstream T-rich elements) are well-positioned with respect to the new CSE. Second, we find another group of variants with dampened effect sizes when mutating the canonical CSE into a weaker form (Fig. 4D, Additional file 1: Fig. S4B). Rather than redundant CSE motifs, these PASs contain many well-positioned auxiliary motifs (CFIm- and CstF-binding motifs and T-rich elements) which dampen the loss of the canonical CSE hexamer. This hypothesis agrees with earlier work suggesting that weak CSEs are efficient polyadenylation elements when found in a strong sequence context [61, 62]. To validate this phenomenon directly in the data, we compared the measurements of all CSE mutations from the MPRA and found a significantly lower variant effect size in PASs with redundant CSE hexamers (Fig. 4E; Additional file 1: Fig. S4C).

### Functional variant predictions correlate with human APA QTLs

**Fig. 5:**
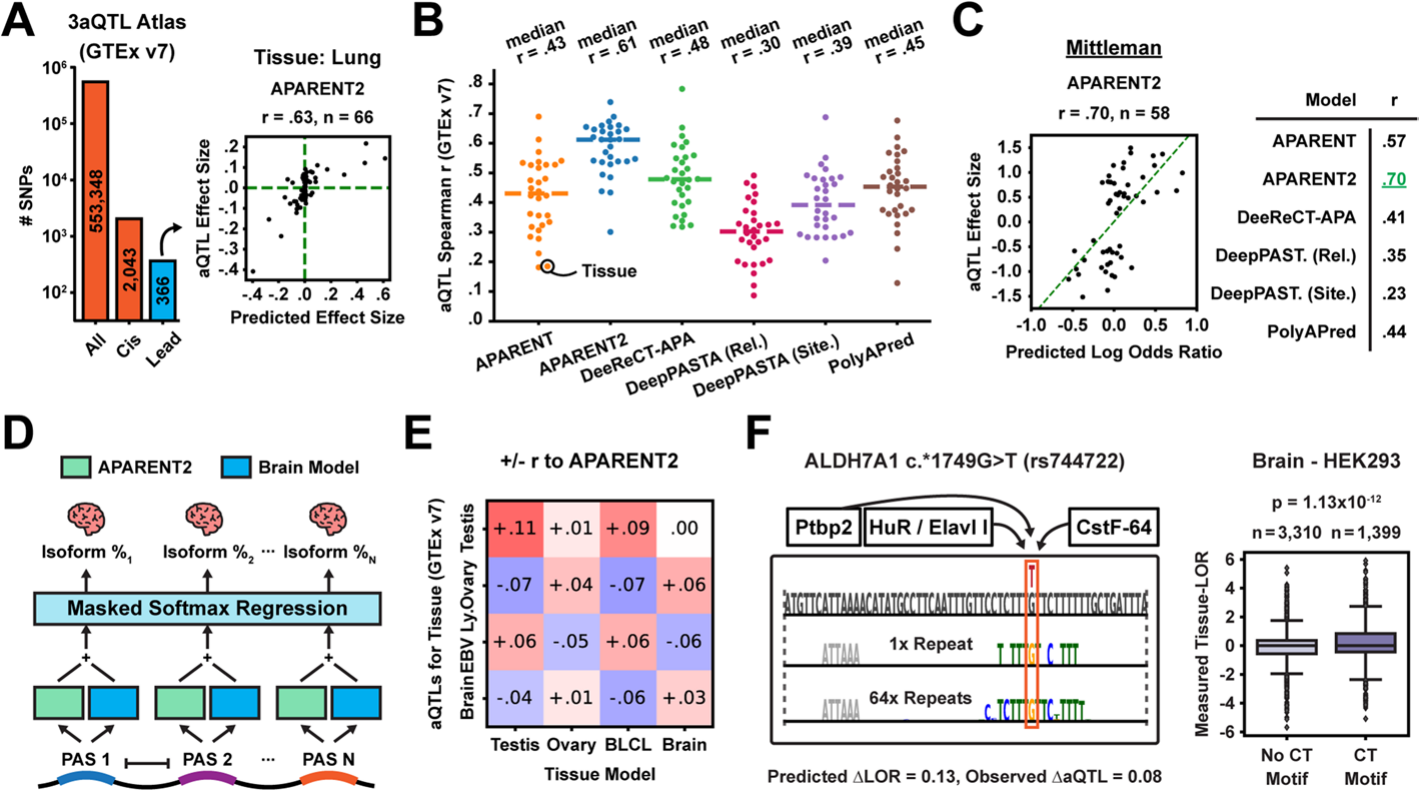
Inferring 3′ aQTL effect sizes from sequence. **A** Total number of 3′ aQTLs, cis-acting aQTLs, and lead aQTLs respectively (GTEx v7). Right: Predicted vs measured aQTL effect sizes in the lung. **B** Predicted vs measured 3′ aQTL effect size Spearman *r*’s (GTEx v7). Each dot corresponds to the correlation in a particular tissue type. **C** Predicted vs measured aQTL effect sizes of the data from Mittleman et al. [21] ($n = 58$). **D** Multiple softmax regression for predicting tissue-specific isoform abundance. APARENT2 (green) and the tissue model (blue) are used to score each PAS. **E** Increase (red) or decrease (blue) in Spearman *r* when using a particular tissue model to scale the 3′ aQTL predictions made by APARENT2 in a given GTEx tissue (testis, ovary, B-cell lymphocytes, and brain). **F** Reconstructive mask for a SNP in the ALDH7A1 gene, with a brain-specific effect. The bottom mask is the result of 64 randomly initialized optimization attempts. Boxplot shows differential PAS usage in data from Lianoglou et al. [42]

To assess the APA models on variant prediction within a native genomic context, we downloaded the recently published atlas of APA QTLs (3′ aQTLs) from GTEx v7 [20]. The majority of aQTL measurements involve distant SNPs far away from any PAS, which is beyond the scope of APARENT2. We thus narrowed the data to the subset of variants that occur close enough to the core hexamer of an annotated PAS in PolyADB (within 50nt; *n* = 2043). We further filtered the data on lead SNPs (the most significant SNP for a given APA event), resulting in a total of 366 3′ aQTLs measured among 44 tissue types (Fig. 5A). We then tasked each model with inferring the aQTL effect size due to each variant (Fig. 5A, B, Additional file 1: Fig. S5A-B). APARENT2 had the highest median correlation across all tissues (Spearman *r* $= 0.61$) and was followed by DeeReCT-APA (*r* $= 0.48$). We obtained similar results when replicating the analysis on 1223 lead SNPs from the newer GTEx v8 atlas [41] (Additional file 1: Fig. S5C-E). We further benchmarked the models on a separate 3′ aQTL dataset [21], consisting of 58 SNVs occurring near annotated PASs among 52 HapMap Yoruba human lymphoblastoid cell lines (Fig. 5C, Additional file 1: Fig. S5F). APARENT2 again were the most correlated with the measured effects (*r* $= 0.70$). Finally, by comparing our variant predictions to 1007 intronic GTEx eQTLs and 2225 3′ UTR eQTLs [63], we validated an observation made by Mittleman et al. [21] that mRNA expression is significantly downregulated due to gain-of-function mutations in intronic PASs, possibly due to aberrant transcript truncation (Additional file 1: Fig. S5G; across all GTEx tissues, we found that variant effects predicted by APARENT2 in weak intronic PASs had a median negative correlation of $r = -0.3$ compared to the measured eQTL effect sizes).

### Tissue-specific variant prediction as residual learning

The 3′ aQTL effect sizes above are tissue-specific, yet APARENT2’s predictions are not. The reason we observe high correlation is because APA, for most genes and PASs, is not differentially regulated [64]. Thus, predictions of APARENT2, which was trained on MPRA data from HEK293 cells, correlate quite well across all tissues. However, we asked whether we could improve variant predictions on some of the aQTLs by combining native, tissue-specific 3′-end sequencing data with the single-cell line MPRA data in a hybrid model. Here, we draw inspiration from earlier work by Cheng et al. [65], where tissue-specific splicing models were used to scale the variant predictions of a non-tissue-specific model. This hybrid approach is motivated by the idea that the non-tissue-specific model, which has been trained on a large MPRA, can provide more accurate baseline predictions. The tissue-specific models, then, are used only to predict residual up- or downregulation due to tissue-specific *trans*-acting regulators and their cognate cis-acting motifs.

We focused on 4 human tissues and cell types that have previously been reported to exhibit differential polyadenylation [64]: testis, ovary, B-cell lymphocytes (BLCL), and brain. We downloaded publicly available 3′-end sequencing data for HEK293, testis, ovary, BLCL, and brain [42] and mapped the RNA-Seq reads to annotated PASs in APADB [57]. In total, we collected APA isoform data for 6440 genes, each gene having between 2 and 10 PASs. First, in agreement with earlier studies suggesting that weaker PASs are upregulated in the testis, we observed that the APARENT2 PAS score itself is predictive of differential usage in the testis; the isoform odds ratio increases ${\sim }1.5$-fold in the testis for proximal PASs with scores ${<}0$ and distal competing signals with scores ${>}0$ ($p = 2.5{\times }10^{-50}$; Additional file 1: Fig. S5H). Next, using these data, we trained four tissue-specific models to learn the residual APA regulation necessary to predict tissue-specific differences superimposed on the baseline APARENT2 predictions (Fig. 5D; Spearman *r* $= 0.20$–0.41 on held-out test data, Additional file 1: Fig. S5I). After training, we used each tissue-specific model to scale the GTEx effect size predictions (Additional file 1: Fig. S5J). With this approach, we raised the median aQTL Spearman correlation from 0.66 to 0.72 in these 4 tissues (Fig. 5E, Additional file 1: Fig. S5K-L).

Finally, we applied our mask-based attribution framework to interpret tissue-specific variants on the basis of the residual tissue models. In Fig. 5F, we investigate GTEx SNP rs744722, which has a positive 3′ aQTL effect size in the brain but a median negative effect size in other tissues. Our interpretation suggests that the variant modifies a T/GT/CT-rich motif by removing one of the Gs. We hypothesized that this SNP alters the affinity for CstF binding, which has an overall negative impact in most tissues, but has a net-positive effect in the brain due to the upregulated levels of HuR/Elavl I and Ptbp2, which are RBPs known to compete with CstF binding in T-rich regions [6, 7]. CLIP data suggests that CstF binds overlapping the mutation site in ADH7A1 [66] and we find in the native transcriptomic training data that CT-rich motifs are associated with upregulated PAS usage in the brain ($p = 1.13{\times }10^{-12}$). In Additional file 1: Fig. S5M, we interpret a similar loss-of-CstF binding mutation, which is observed to have a more negative effect size in testis compared to other tissues. Consistent with earlier studies, we find evidence that GT-rich motifs are associated with differential APA in the testis, which is likely due to elevated levels of CstF [67–69].

### Variant prediction across the 3′ UTR by combining models of polyadenylation and mRNA stability

**Fig. 6:**
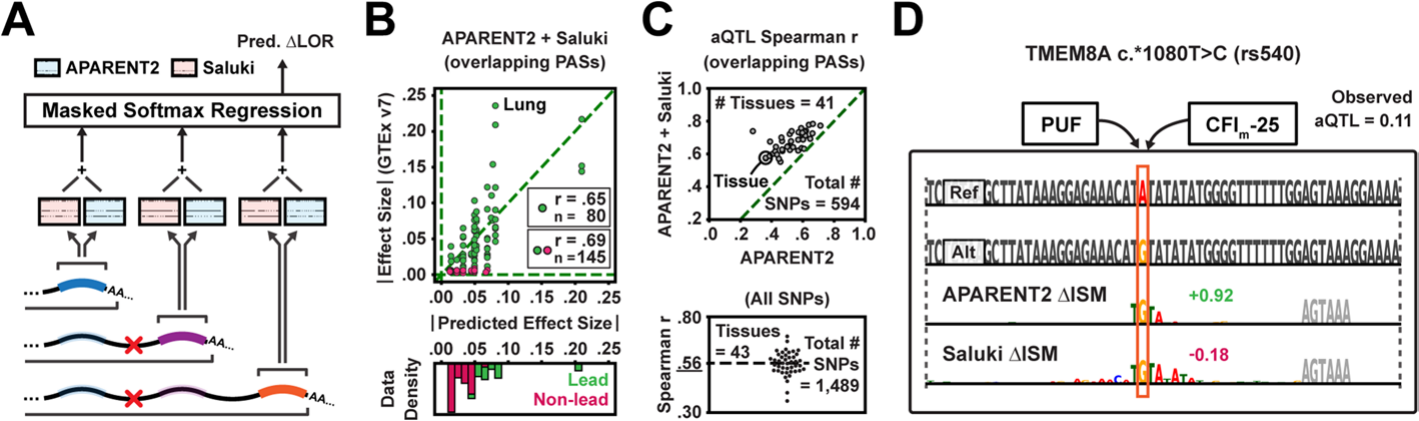
Extending aQTL predictions to the entire 3′ UTR. **A** The total impact of a 3′ UTR mutation on isoform abundance is scored by APARENT2 and Saluki. **B** Absolute value of predicted vs measured 3′ aQTL effect sizes for lead SNPs and a matched set of non-lead SNPs overlapping PASs in the lung (GTEx v7). Final predictions are made by isotonic regression trained on all non-lung SNPs. **C** Top: Predicted vs measured aQTL effect size Spearman *r*’s for SNPs overlapping PASs. Each dot represents the correlation in a given tissue. Bottom: Predicted vs measured effect sizes for all 3′ UTR SNPs, using the joint APARENT2+Saluki model. **D** Difference in ISM maps between mutant and wildtype sequence (rs540). Green/magenta annotations correspond to APARENT2/Saluki predictions

We found in Fig. 1G that modeling both pA efficiency and mRNA isoform stability improved the overall fit to endogenous 3′-end sequencing data. We thus hypothesized that the combined model would also improve aQTL effect size estimation by correcting APARENT2’s predictions for effects on stability (Fig. 6A). We tuned the regression weights of the combined model on tissue-pooled measurements from PolyADB V3 [54] and used the model’s distal-most output to score the impact of SNPs from the GTEx v7 catalog on isoform abundance (Fig. 6B) [20]. When evaluating the combined model on SNPs that overlap both the APARENT2 and Saluki input windows (*n* $= 594$), it consistently outperformed APARENT2 alone for all tissues (Fig. 6C, top, Additional file 1: Fig. S6A-B; median Spearman *r* increased from 0.53 to 0.68). When applied to all lead SNPs occurring anywhere in the 3′ UTR (including outside APARENT2’s window; *n* $= 1489$), the median correlation reached 0.58 (Fig. 6C, bottom).

To exemplify the types of co-localizing regulatory signals of APA and mRNA stability that benefit from joint modeling, we applied in silico saturation mutagenesis (ISM) to the wildtype and mutant sequence surrounding a GTEx SNP (rs540) that was better predicted when using both models (Fig. 6D). The attributions show that both models respond to the creation of a TGTA motif. However, while APARENT2 recognizes this as the putative binding motif for CFIm (a pA enhancer), it neglects the simultaneous creation of a putative Pumilio-binding motif (a de-stabilizing RBP). The effect of this RBP is accounted for by Saluki.

### Disruptive polyadenylation variants are selected against in the human population

**Fig. 7:**
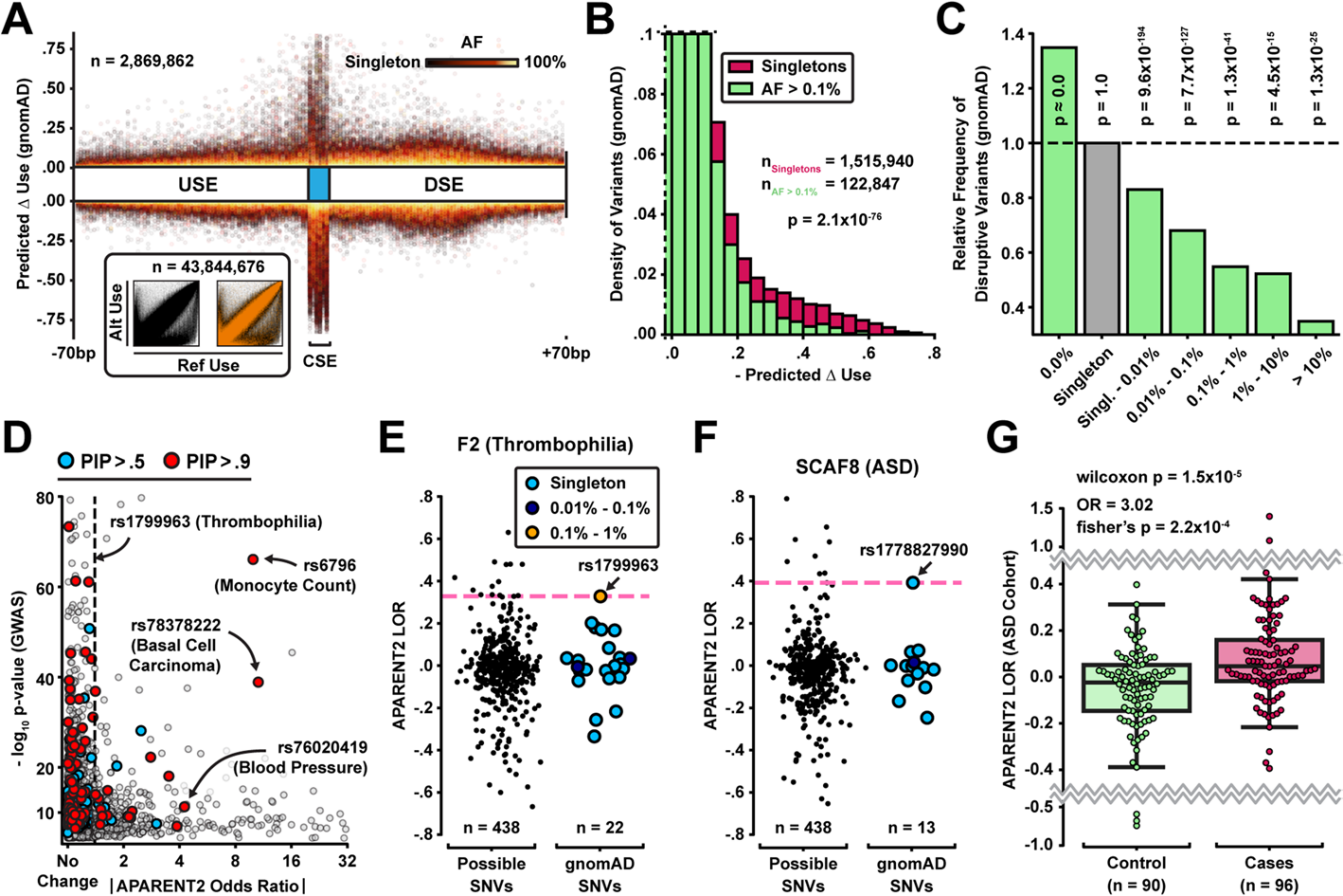
Large-scale analysis of polyadenylation signal mutations and their implication in health and disease. **A** Relative position of mutation vs predicted $\Delta$ isoform abundance for all PAS variants in gnomAD (*n* = 2.8 million). Color intensity represents allele frequency. Inset: Reference vs alternate isoform abundance for all 43.8 million potential PAS SNVs (orange = gnomAD variants). **B** Distribution of predicted $\Delta$ isoform abundance for common gnomAD variants (AF ${>}0.1\%$; green) and singletons (magenta). **C** Relative enrichment of disruptive variants (${\Delta }$isoform abundance $< -0.15$) with respect to singleton variants. Wilcoxon *p*-values are shown above each bar. **D** Absolute predicted isoform fold change vs *p*-value of fine-mapped GWAS SNPs from CAUSALdb ($95\%$ credible set, *n* = 4200) [70]. **E** Distribution of predicted log odds ratios for the F2 PAS. **F** Distribution of predicted log odds ratios for the SCAF8 PAS. **G** Predicted log odds ratios among ASD cases and controls from a WGS study [51]

We next sought to understand the connection between the functional impact of genetic variation on polyadenylation and human health. Using APARENT2, we performed full in silico saturation mutagenesis of every annotated PAS in PolyADB V3 [54] and imputed the effect size (odds ratio) of every possible SNV (*n* ${>} 43.8$ million). For each PAS, we calculated the average wildtype isoform usage across all tissues in PolyADB. We then re-calculated the isoform usage in the presence of each mutation by using the APARENT2 prediction to scale the isoform odds. Given these two quantities, we estimated the change in isoform proportion (${\Delta }$use) due to each variant. When cross-referencing our predictions against the ${>}2.8$ million PAS SNVs curated from the ${>}71,000$ genomes sequenced in gnomAD v3 [50] (Fig. 7A), we found that disruptive loss-of-function variants (resulting in downregulated pA) are depleted in common variants (AF ${>}0.1\%$) compared to singletons (wilcoxon $p = 2.1{\times }10^{-76}$; Fig. 7B, Additional file 1: Fig. S7A). Disruptive loss-of-function variants (${\Delta }$use $< -0.15$) occur ${\sim } 2.5$-fold less frequently among common variants (AF ${>}10\%$) than singletons and they occur ${\sim } 1.4$-fold more frequently in unobserved variants (AF $= 0\%$) compared to singletons (Fig. 7C). These results suggest a negative selection pressure on disruptive variants in human polyadenylation signals.

### Gain-of-function mutations in the 3′-end are associated with clinical conditions

Most of the known deleterious polyadenylation variants are highly disruptive CSE mutations [16–18]. However, while we found in the previous section that highly disruptive loss-of-function variants are generally selected against, they also frequently occur as common variants. This suggests that we cannot use the inferred effect on polyadenylation alone as a predictor for variant pathogenicity. To highlight this phenomenon, we intersected our predictions against CAUSALdb [70], a database containing fine-mapping results from over 3000 GWAS summary statistics (including UK Biobank [71] and GWAS Catalog [72]). We first noticed that SNPs with a large posterior inclusion probability (PIP) from UK Biobank are enriched for disruptive APA variants (Additional file 1: Fig. S7B) [73]. We then identified 96 SNPs with PIP ${>}90\%$ and many of these are known deleterious variants that act through APA (Fig. 7D). As expected, the predicted effect size of these known APA mutations varies considerably. For example, the variant rs1799963 in the F2 gene increases pA efficiency only modestly (${<}1.5$-fold) but is responsible for thrombophilia [19]. In contrast, the cancer-associated variant rs78378222 disrupts the PAS of the TP53 gene ${>}10$-fold. Clearly, the downstream consequence of disrupted polyadenylation depends on the importance of the affected APA isoforms, not to mention the gene itself. However, we can assume that a mutation is likely not deleterious if it occurs in a PAS with common variants that have even larger effect sizes. Thus, we can eliminate PAS mutations and classify them as likely benign when they co-occur with putative functional common variants in gnomAD with high impact on polyadenylation. For example, the pathogenic variant rs1799963 would not be eliminated, since it is the variant with the largest predicted odds ratio of all observed F2 variants in gnomAD (Fig. 7E).

Using the stratification process above, we investigated the link between misregulated polyadenylation and autism spectrum disorder (ASD), a relationship which has been suggested before but mainly at the trans-regulatory level and less in terms of cis-regulatory variation in the 3′ UTR [74–77]. Figure 7F displays an example rare variant (rs1778827990) associated with ASD [78]. The suspected variant has a considerably higher (positive) effect size than any of the observed variants in gnomAD. Hypothesizing that gain-of-function mutations may be linked to ASD, we ran APARENT2 on whole-genome sequencing (WGS) data from 1902 families [51, 79] and found that variants overlapping PASs in cases are enriched for gain-of-function compared to controls (Wilcoxon $p = 0.049$, $n_{\text {cases}} = 297$, $n_{\text {controls}} = 296$). When removing variants that co-occur with higher-impact common SNPs in gnomAD (AF ${>}0.01\%$), the significance increased (Wilcoxon $p = 2.1{\times }10^{-4}$), and when also removing variants that occur in PASs with a protective downstream PAS within 200nt, the significance increased further (Wilcoxon $p = 1.5{\times }10^{-5}$; see Section 5 for filtering procedure) (Fig. 7G, Additional file 1: Fig. S7C). We observed a 3.02-fold enrichment of gain-of-function mutations in cases (Fisher’s $p = 2.2{\times }10^{-4}$). As additional validation, the predicted effect sizes of variants from the control set were indistinguishable from variants in gnomAD after applying the same filtering (Wilcoxon $p = 0.341$) while case variants were significantly different (Wilcoxon $p = 2.7{\times }10^{-5}$). Finally, we found an enrichment among PAS case variants of the gene ontology terms’ regulation of primary metabolic process’ (FDR = $6.79{\times }10^{-02}$) and “protein binding” (FDR = $3.48{\times }10^{-04}$) [80]. We found no significant enrichment among controls.

When replicating the analysis against a smaller WGS study of 200 families [78], we again observed an enrichment of gain-of-function mutations in cases relative to the controls from An et al. [51], but the results were only significant with less stringent filtering criteria (Wilcoxon $p = 0.039$; Additional file 1: Fig. S7D-E). The predicted effect sizes of case variants were not significantly different from gnomAD variants (Wilcoxon $p = 0.127$), but the trend was similar to that of the larger cohort data so this is likely due to insufficient sample size. Even in this smaller cohort, we can use APARENT2 to functionally interpret variants with high predicted effect sizes. For example, the variant highlighted in Fig. 7F (rs1778827990) is predicted to be a putative gain-of-CstF binding mutation (Additional file 1: Fig. S7F).

### An MPRA of clinically relevant variants in multiple cell lines

**Fig. 8:**
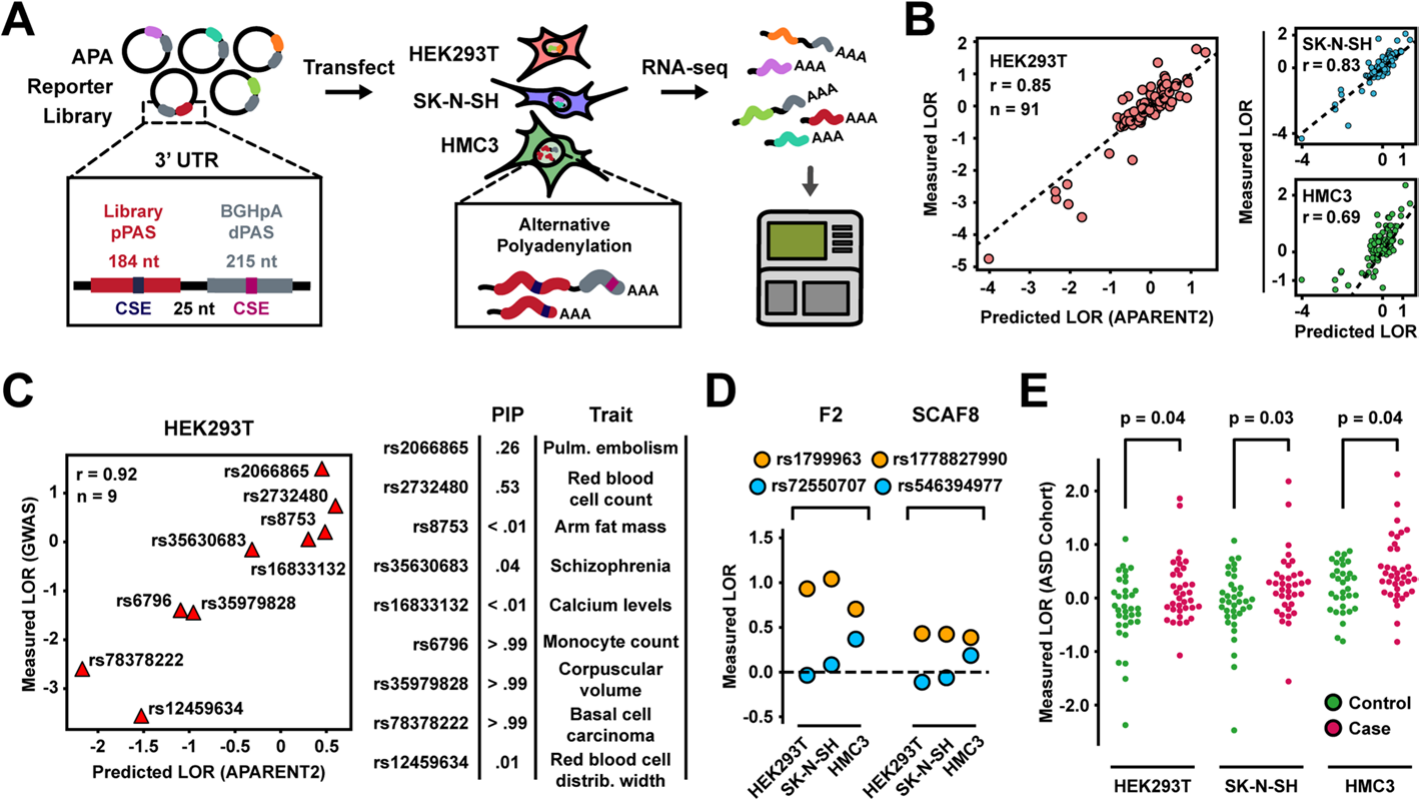
MPRA validation in multiple cell lines. **A** APA reporter system for measuring variant effects in HEK293T, SK-N-SH and HMC3. **B** Predicted vs measured variant effects (LORs) in the three assayed cell lines. **C** Predicted vs measured effects of 9 GWAS SNPs (PIP = posterior inclusion probability). **D** Measured effects of 2 SNVs in the F2 and SCAF8 genes (orange), alongside common gnomAD SNPs (blue). **E** Measured effects of 76 autism variants from An et al. [51]. *p*-values are computed with two-sided Wilcoxon tests

To validate the predictions made for clinically relevant variants and to assess brain-specific effects, we experimentally tested 94 PAS SNVs in a plasmid reporter MPRA measured in HEK293T, SK-N-SH, and HMC3 cells (Fig. 8A; Additional file 1: Fig. S8A-C). These variants consisted of 38 case and 38 control variants from the ASD data set (19 variants with the largest positive effects and 19 variants with the largest negative effects for both cases and controls, after removing variants that occur in PASs with higher-impact common SNPs in gnomAD; AF ${>}0.01\%$), in addition to 9 GWAS SNPs with diverse predicted effects and other disease-relevant examples in the F2 and SCAF8 genes. APARENT2’s predictions agreed well with the measurements in HEK293T and SK-N-SH (Fig. 8B; *r *= 0.85 in HEK293T and 0.83 in SK-N-SH) but were less concordant with HMC3 (*r* = 0.69), suggesting microglia-specific PAS usage modulation.

APARENT2 could accurately predict the effects of the selected GWAS SNPs (*r* $= 0.92$ in HEK293T; Fig. 8C). However, the chosen variants that displayed loss of function (the majority of which were associated with cardiovascular traits) had less extreme effects in HMC3 (Additional file 1: Fig. S8D). Additionally, we validated the effects of rs1799963 (F2; thrombophilia) and rs1778827990 (SCAF8; autism case) and found that these variants behave similarly across cell lines (Fig. 8D). Finally, we recapitulated a significant enrichment of gain-of-function variants in autism cases for all cell lines (Fig. 8E, Additional file 1: Fig. S8E; Wilcoxon $p \le 0.04$). There were ${\sim }3$-fold more case variants with effect sizes larger than controls in HMC3 than there were in HEK293T and SK-N-SH, suggesting they have potential microglia-specific effects.

## Discussion

In this paper, we developed an improved human polyadenylation variant prediction model, APARENT2, based on deep residual neural networks. We systematically compared this model to other sequence-predictive APA models, including the original APARENT network, on the task of predicting functionally disruptive variants from MPRA data and human APA QTLs. We found that APARENT2 was considerably better at variant effect size estimation compared to other models, in particular for cryptic variants outside of the CSE. We further trained tissue-specific residual models for the testis, ovary, B-cell lymphocytes, and brain and used these to improve variant prediction in human tissues. By combining rich modeling with mask-based attribution, we extracted complex cis-regulatory rules and elucidated cooperativity among core polyadenylation signal motifs. For example, we found super-additive interactions between the CFIm-binding motif TGTA and downstream AT-rich elements. Conversely, we identified protective buffering effects of redundant and well-positioned core hexamers that can “take over” in case the original CSE is disrupted by mutations.

An intriguing finding of our work is that the same PAS scoring function accurately predicts relative isoform abundance in multi-PAS genes and absolute transcript levels in genes containing a single PAS. These results are consistent with a simple model of polyadenylation where a PAS emerging during transcription is used with an independent probability that is determined entirely by the sequence of that signal. If an additional PAS occurs in the emerging transcript, its usage is again determined independently by the sequence. Moreover, 3′-end processing via cleavage and polyadenylation is in competition with other processes such as RNA degradation and transcriptional feedback that reduce mature mRNA levels. By incorporating a model of RNA stability with APARENT2, we further improved APA QTL predictions by correcting for differential isoform stability.

We applied APARENT2 to make functional predictions on 44M PAS variants in the human genome, orders of magnitude more than would currently be possible even in a high-throughput reporter assay. Moreover, unlike statistical methods such as aQTL analysis, functional predictions can be made even for variants that have not yet been observed, but may well occur, in the human population. Finally, we combined APARENT2’s variant predictions with additional evidence from a large variation database (gnomAD). This allowed us to enrich our predictions by disregarding mutations that co-occur in PASs with common high-impact variants, as these PASs are likely not important for function. Using this approach, we found a ${\sim }3$-fold enrichment of gain-of-function variants (leading to more efficient pA) in individuals with autism spectrum disorder.

It is important to note that we cannot definitively classify mutations in PASs with high predicted effect size as causative of autism; both loss- and gain-of-function variants occur frequently in controls, suggesting many gain-of-function variants in cases are likely benign. However, the significant over-representation of gain-of-function mutations in cases suggests that *some* of those variants contribute to autism. We experimentally validated predicted high-impact variants in an MPRA and showed that case variants indeed are enriched for gain-of-function, with more extreme effects in microglia-derived HMC3 cells. These results signify the importance of having a functional model; the number of mutations occurring in PASs was almost identical between cases and controls and we only discovered the signal in APARENT2’s predictions.

## Conclusions

The sequence-to-function model developed here, APARENT2, enabled more accurate variant effect predictions within human polyadenylation signals than any previous model. Importantly, the deep residual network architecture employed is interpretable; by applying mask-based attribution techniques, we could project higher-order features of the polyadenylation code down to the sequence and validate these interactions using functional variant effect measurements. This approach led to the discovery of multiple epistatic interactions between cis-regulatory RNA binding protein motifs. By computationally assessing the impact of every potential polyadenylation signal mutation in the genome and intersecting these predictions against human variation data, we could draw powerful associations to phenotypic traits and clinical conditions.

## Methods

### Neural network architecture

APARENT2 is based on residual blocks of dilated convolutions [52] and is architecturally similar to the SpliceAI model [27]. Let $\mathcal {P}$ be the APARENT2 model. As input, $\mathcal {P}$ receives a one-hot coded sequence $\varvec{x} \in \{0, 1\}^{205 \times 4}$, which represents the proximal PAS, and a one-hot coded variable $\varvec{l} \in \{0, 1\}^{13}$ which indicates the source 3′ UTR sub-library from the MPRA training data [36]. Internally, $\mathcal {P}$ consists of 7 *residual groups*, and each residual group is made up of 4 *residual blocks*. A residual block (Additional file 1: Fig. S1A) consists of two batch-normalized, ReLU-activated one-dimensional convolutional layers with a specific filter dilation rate. Each block also has a skip connection, which mathematically performs an unweighted element-wise addition. Each residual group consists of residual blocks of the same dilation rate. For this particular network, the 7 residual groups use the following sequence of dilation rates: 1, 2, 4, 8, 4, 2, 1. Between each residual group, there is an extra skip connection to the final output layer. Only $\varvec{x}$ is passed through the series of residual blocks, producing in the end a single-channel vector of non-normalized cleavage scores $\varvec{s}(\varvec{x}) \in \mathbb {R}^{206}$ (Note that $\varvec{s}$ has one position more than $\varvec{x}$; this extra position represents the total isoform score of the distal signal). The library indicator variable $\varvec{l}$ is multiplied with a position-specific weight matrix $\varvec{W} \in \mathbb {R}^{206 \times 13}$ and linearly combined with $\varvec{s}(\varvec{x})$, producing new scores $\hat{s}(\varvec{x}, \varvec{l})_{j} = s(\varvec{x})_{j} + \sum \nolimits _{k=1}^{13} w_{jk} \times l_{k}$ ($1 \le j \le 206$) which have effectively been scaled with a library-specific intercept. Finally, $\mathcal {P}$ produces a normalized 206-way cleavage distribution $\hat{\varvec{y}} \in [0, 1]^{206}$ by applying the softmax transform (Eq. 1).

1 $$\begin{aligned} \hat{y}_{j} = \mathcal {P}(\varvec{x}, \varvec{l})_{j} = \frac{e^{\hat{s}(\varvec{x}, \varvec{l})_{j}}}{\sum \nolimits _{k=1}^{206} e^{\hat{s}(\varvec{x}, \varvec{l})_{k}}} \end{aligned}$$

All residual blocks in APARENT2 have 32 channels and all convolution filters are 3 positions wide. Note that there is no explicit sigmoid output representing the total proximal isoform proportion. Rather, the proximal isoform proportion is computed as the sum of cleavage probability mass 7–57 nt downstream of the start of the proximal CSE (which is located at position 70): $\hat{y}_{\text {iso}} = \sum \nolimits _{j=77}^{127} \hat{y}_{j}$. However, for some variant prediction tasks, the proximal isoform is defined as “any cleavage that is not distal” (i.e., the data processing of those datasets considered cleavage from nearby competing cryptic PASs as “proximal”). In that case, we define the predicted proximal isoform proportion as $\hat{y}_{\text {iso}} = \sum \nolimits _{j=1}^{205} \hat{y}_{j}$ (and $\hat{y}_{206}$ is the distal proportion).

### MPRA training data

The MPRA dataset from Bogard et al. [36] was re-processed to make the training data more uniform. First, the original dataset consisted of 185-nt-long sequences, starting 50 nt upstream of the proximal CSE. However, for some of the sub-libraries (the MPRA consisted of 13 sub-libraries with different 3′ UTR contexts), an additional random barcode was located from 70 to 50 nt upstream of the CSE. In the re-processed version of the data, we included 20 nt of additional sequence upstream of the CSE to capture these barcodes.

Second, for some of the sub-libraries, the exact cleavage distributions were not estimated from the RNA-Seq data. Instead, these sub-libraries only included total proximal-to-distal isoform proportions. We re-mapped the RNA-Seq reads to these sub-libraries and augmented the data with the missing cleavage distributions.

Finally, the original models were only trained on about 2.4 million of the degenerate (randomized) MPRA data (3 of 12 sub-libraries of the random MPRA were held out for independent testing), and it was not trained on any of the assayed human APA sites from the designed MPRA. Here, we trained the network on data from all of the degenerate sub-libraries, resulting in 3.3 million training sequences and 80,000 sequences for validation and testing each. We also included human intronic PAS sequences from the designed MPRA (which had been measured in a 3′ UTR reporter), adding approximately 10,000 additional high-quality measurements to the training data. To keep the variant prediction results unbiased, we did *not *train the network on any of the human 3′ UTR sequences from the variant MPRA. Note that, as in the original paper, MPRA sequences with ${>}75\%$ adenine bases in a 12–20-bp region were removed to minimize internal priming artifacts [36]. Hence, the resulting trained model cannot be used on sequences with long adenine stretches.

### Cleavage and isoform cost function

Given the training data $\mathcal {D} = \{\varvec{x}^{(i)}, \varvec{y}^{(i)}\}_{i=1}^{N}$, where $\varvec{x} \in \{0, 1\}^{205 \times 4}$ is a one-hot coded representation of the proximal (degenerate) polyadenylation signal and $\varvec{y} \in [0, 1]^{206}$ is the measured 3′ cleavage distribution, we trained APARENT2 to minimize the hybrid cost function given in Eq. 2.

2 $$\begin{aligned} \mathcal {L}_{\text {train}}(\{\varvec{x}^{(i)}, \varvec{y}^{(i)}\}_{i=1}^{N}) = \frac{1}{N} \sum \limits _{i=1}^{N} \left( KL\left[ \hat{\varvec{y}}^{(i)} || \varvec{y}^{(i)} \right] + KL\left[ \hat{y}^{(i)}_{\text {iso}} || y^{(i)}_{\text {iso}} \right] \right) \end{aligned}$$

Here, $KL\left[ \hat{\varvec{y}}^{(i)} || \varvec{y}^{(i)} \right]$ is the KL divergence between the predicted cleavage distribution $\hat{\varvec{y}}^{(i)} = \mathcal {P}(\varvec{x}^{(i)}, \varvec{l}^{(i)})$ and measured distribution $\varvec{y}^{(i)}$ (defined in Eq. 3). $KL\left[ \hat{y}^{(i)}_{\text {iso}} || y^{(i)}_{\text {iso}} \right]$ is an extra consistency term used to fit the sum of a subset of softmax outputs $\hat{y}_{\text {iso}} = \sum \nolimits _{k=80}^{110} \hat{y}_{k}$ to the total observed proximal isoform proportion $y_{\text {iso}} = \sum \nolimits _{k=80}^{110} y_{k}$ (defined in Eq. 4). We found empirically that the extra consistency term produced better isoform fold-change predictions for downstream variant prediction tasks, presumably by increasing the importance of predicting cleavage accurately within the region specified by this loss term (which is where the majority of proximal cleavage occurs for most PASs). Note that the last position in the target vector ($y^{(i)}_{206}$) corresponds to the total isoform proportion (total cleavage) of the distal (non-degenerate) PAS.

3 $$\begin{aligned} KL\left[ \hat{\varvec{y}} || \varvec{y} \right]&= \sum \limits _{k=1}^{206} y_{k} \times \log \left( \frac{y_{k}}{\hat{y}_{k}}\right) \end{aligned}$$

4 $$\begin{aligned} KL\left[ \hat{y}_{\text {iso}} || y_{\text {iso}} \right]&= y_{\text {iso}} \times \log \left( \frac{y_{\text {iso}}}{\hat{y}_{\text {iso}}}\right) + (1 - y_{\text {iso}}) \times \log \left( \frac{1 - y_{\text {iso}}}{1 - \hat{y}_{\text {iso}}}\right) \end{aligned}$$

### Model training

We trained APARENT2 for 5 epochs with mini-batch SGD using the Adam optimizer in Keras [81, 82] with default parameters and batch size $= 64$. During training, we randomly shift both the input sequence $\varvec{x}^{(i)}$ and target cleavage distribution $\varvec{y}^{(i)}$ by at most 15 nt in either direction (Additional file 1: Fig. S1B). As such, the CSE position (which all sequences are initially aligned against) varies during training, forcing the network to learn to displace the cleavage distribution according to the location of the CSE. This helped the network give better predictions to locally competing CSE hexamers in the nearby USE or DSE regions.

### Web tool

We developed a web tool for running in silico saturation mutagenesis across human polyadenylation signals from the PolyADB V3 data [54] (Additional file 1: Fig. S9). The application loads a graph tool based on D3.JS [83], where predicted cleavage distributions can be explored interactively. *Note:* This web application has been online since 2019, but has been relying on the original APARENT model for predictions.

### Endogenous datasets

We collected four different sets of human 3′-end sequencing data in order to benchmark the APA models at the tasks of predicting pairwise and multi-PAS isoform proportions. We first downloaded the tissue-pooled version of APADB [57] from http://tools.genxpro.net:9000/apadb/download/track/hg19.apadb_v2_final.bed/ (dataset # 1). We obtained the RNA-seq counts of Lianoglou et al. [42] from https://cbio.mskcc.org/leslielab/ApA/atlas/ and mapped the read positions to the annotated PASs in APADB. From the mapped cleavage position counts, we estimated the total read count $c_{i}^{(k)}$ that support APA isoform *i* of gene *k* aggregated over all tissues (dataset # 2). Similarly, the aggregated isoform counts from Derti et al. [13] were downloaded from GEO (accession GSE30198) and mapped to the PAS sequences from APADB (dataset # 3). The final dataset consisted of the tissue-pooled RNA-seq counts from PolyADB V3 [54] and was downloaded from https://exon.apps.wistar.org/PolyA_DB/v3/download/3.2/human_pas.zip (dataset # 4).

For the task of pairwise APA isoform prediction, we collected pairs of adjacent 3′ UTR PASs with a total read count ${\ge }500$. The two sites had to be at least 100bp apart and at most 4000bp apart. Finally, sequences with more than 7 consecutive adenine bases were removed to minimize the risk of internal priming. For the multi-PAS prediction task, we kept genes with at least 2 annotated PASs in APADB (or PolyADB) and at most 10 PASs. We removed genes with less than 10 total counts, or with PASs separated by less than 50bp or more than 40,000bp. Genes with PASs that contain more than 13 consecutive adenines were removed.

### Variant datasets

We benchmarked the APA models on two 3′ UTR MPRAs, two native transcriptomic 3′ aQTL datasets, and GWAS data. The specific data filters and measurements of each dataset are described below.

***Isoform MPRA:*** [36] This APA variant MPRA contains SNVs near PASs of disease-implicated 3′ UTRs from ClinVar, HGMD, or ACMG genes [38–40]. We filtered the data to include only variants where the wildtype and variant sequences each had a mean unique UMI read count $>200$ from at least 5 barcoded replicates. This resulted in a total of 12,350 retained variants. We estimated the log odds ratio (log fold change) $\text {LOR}(y_{\text {wt}}, y_{\text {var}})$ of each variant’s proximal isoform abundance $y_{\text {var}}$ with respect to the wildtype abundance $y_{\text {wt}}$ (Eq. 5). These isoform abundances were calculated by summing all cleavage probabilities mapping to cut sites $+0$ to $+50$ nt downstream of the CSE.

5 $$\begin{aligned} \text {LOR}(y^{(\text {wt}}, y^{(\text {var})}) = \log \left( \frac{y^{(\text {var})} / (1 - y^{(\text {var})})}{y^{(\text {wt})} / (1 - y^{(\text {wt})})}\right) \end{aligned}$$

In one of the benchmarks, we compared the model performances of classifying disruptive APA variants. A variant was deemed “disruptive” if the absolute value of its isoform odds ratio with respect to the wildtype abundance was larger than 2: $\text {Disruptive} = 1 \text { if } \left| \frac{\hat{y}_{\text {var}} / (1 - \hat{y}_{\text {var}})}{\hat{y}_{\text {wt}} / (1 - \hat{y}_{\text {wt}})} \right| > 2 \text { else } 0$.

The dataset was downloaded from https://github.com/johli/aparent.

***Expression MPRA:*** [35] This 3′ UTR MPRA measured the RNA/DNA fold changes of viral PAS variants. We filtered the data to include only sequences that were in the test set of the PolyApredictor model. We also removed sequences that contained either a stretch of at least 10 consecutive A’s, or sequences containing the subsequence “AGA” at position 41, as the measurements of these sequences seemed to be influenced by artifacts. Finally, we only considered the subset of sequences that were part of the scanning mutagenesis experiments, resulting in a total of 442 variants. For these sequences, we matched the wildtype and variant PASs in order to calculate the RNA/DNA fold change ratio $FCR(u_{\text {wt}}, u_{\text {var}}) = u_{\text {var}} - u_{\text {wt}}$ due to each variant. Here, *u* is the logarithm of the RNA/DNA fold change of a particular sequence.

The model PolyApredictor predicts the log fold changes $\hat{u}$ directly. For all other models, we approximate $\hat{u}$ with the predicted isoform log odds: $\log \left( \hat{y} / (1 - \hat{y}) \right)$. Furthermore, since both PolyApredictor and APARENT2 support cleavage predictions at base-pair resolution, we also compared them on their ability to infer the RNA/DNA fold change ratios $FCR_{j}$ of each variant across every wildtype cleavage position *j*.

The dataset was downloaded from https://github.com/segallab/PolyApredictors.

***GTEx 3′ aQTLs:*** [20, 41] The GTEx v7 3′ aQTL data was downloaded and mapped to the PolyADB V3 annotation [54]. The data was further filtered to only include lead SNPs occurring within 50nt of the most likely CSE of the annotated PAS. This resulted in 366 SNPs with measured 3′ aQTL effect sizes among 44 GTEx tissue types. To predict effect sizes, we first used each model to infer the SNP log odds ratio $\text {LOR}(\hat{y}^{(\text {wt})}, \hat{y}^{(\text {var})})$, which is an estimate of the local effect that the given variant has on the efficiency of the overlapping PAS. Next, given the observed polyadenylation distal usage index $y_{\text {PDUI}} \in [0, 1]$ of a particular PAS averaged across all GTEx tissues and samples, we inferred the SNP effect size $\Delta y_{\text {PDUI}}$ by scaling the measured PDUI with the predicted variant odds ratio (Eq. 6).

6 $$\begin{aligned} \Delta y_{\text {PDUI}} = \frac{1}{\left( 1 + e^{-\lambda \times \text {LOR}(\hat{y}^{(\text {wt})}, \hat{y}^{(\text {var})})} \times (1 - y_{\text {PDUI}}) / y_{\text {PDUI}} \right) } - y_{\text {PDUI}} \end{aligned}$$

In the above equation, $\lambda$ is assigned $+1$ or $-1$ and indicates whether the particular variant is near the distal PAS or not. If the variant is far away from the distal PAS, it is assumed that it acts through a competing PAS and hence the predicted effect is inverted by assigning $\lambda = -1$. We used a simple heuristic to assign $\lambda$: If the SNP is within 150bp from the annotated transcript end, we let $\lambda = +1$, otherwise $\lambda = -1$.

The dataset was downloaded from https://doi.org/10.7303/syn22236281.

*Note:* In Additional file 1: Fig. S5C-E, we replicated the 3′ aQTL benchmark on the newer GTEx v8 atlas. The filter criteria were identical to the GTEx v7 atlas, except for a maximum *p*-value threshold of $10^{-12}$ that was imposed on lead SNPs to increase the quality of the estimated effect sizes. We also assigned $\lambda = +1$ if a SNP occurs within 500bp of the transcript end but creates a canonical de novo CSE hexamer, as such variants were annotated as belonging to the distal isoform.

The dataset was downloaded from https://wlcb.oit.uci.edu/3aQTLatlas.

***HapMap Yoruba lymphoblastoid 3′ aQTLs:*** [21] The 3′ aQTLs were mapped against the PolyADB V3 annotation [54] and narrowed to the subset of SNPs occurring within 50nt of the most likely CSE of each annotated PAS. This resulted in 58 variants measured among 52 HapMap Yoruba human lymphoblastoid cell lines. We used the effect sizes estimated from nuclear mRNA only. The predicted log odds ratio $\text {LOR}(\hat{y}^{(\text {wt})}, \hat{y}^{(\text {var})})$ of each model was directly compared to the 3′ aQTL effect sizes.

The raw data and annotations were available at GEO under accession GSE138197. The processed data, including the estimated 3′ aQTL effect sizes, were provided to us by the authors.

***Fine-mapped GWAS SNPs:*** [70, 73] The GWAS SNPs from the $95\%$ credible set of CAUSALdb [70] were intersected against 3′ UTR PASs from PolyADB V3 [54]. In addition, all fine-mapped GWAS SNPs from the UK Biobank cohort (generated by Kanai et al. [73]), including variants outside the credible set, were downloaded. The predicted log odds ratio $\text {LOR}(\hat{y}^{(\text {wt})}, \hat{y}^{(\text {var})})$ of APARENT2 was compared to the *p*-values and posterior inclusion probabilities of each dataset.

CAUSALdb was downloaded from http://www.mulinlab.org/causaldb/index.html. The UKBB fine-mapping data was downloaded from https://www.finucanelab.org/data.

### Variant prediction models

Following is a list of the APA models that were included in the variant prediction benchmark, with a detailed description of how each model was used and where each model was downloaded from.

***APARENT2:*** (This paper) The model takes as input a 205-nt one-hot coded sequence $\varvec{x} \in \{0, 1\}^{205 \times 4}$ and a MPRA sub-library indicator $\varvec{l} \in \mathbb {R}^{13}$. The model predicts a 3′ cleavage distribution $\hat{\varvec{y}} \in \mathbb {R}^{206}$ ($\hat{y}_{206}$ corresponds to total isoform cleavage). When using the model for variant prediction, we set $l_{11} = 1$ (the human intronic PAS sub-library intercept). The variant log odds ratio $\text {LOR}(\hat{y}^{(\text {wt})}, \hat{y}^{(\text {var})})$ is calculated from a subset of the cleavage outputs. For the MPRA of Bogard et al. [36] and the 3′ aQTLs of Mittleman et al. [21], we define $\text {LOR}(\hat{y}^{(\text {wt})}, \hat{y}^{(\text {var})}) = \text {LOR}(\sum _{j=77}^{127} \hat{y}^{(\text {wt})}_{j}, \sum _{j=77}^{127} \hat{y}^{(\text {var})}_{j})$. For the MPRA of Slutskin et al. [35] and the GTEx 3′ aQTLs [20], we define $\text {LOR}(\hat{y}^{(\text {wt})}, \hat{y}^{(\text {var})}) = \text {LOR}(\sum \nolimits _{j=1}^{205} \hat{y}^{(\text {wt})}_{j}, \sum \nolimits _{j=1}^{205} \hat{y}^{(\text {var})}_{j})$.

***APARENT:*** [36] The original APARENT model, which takes as input a 185-nt one-hot coded sequence $\varvec{x} \in \{0, 1\}^{185 \times 4}$, a MPRA sub-library indicator $\varvec{l} \in \mathbb {R}^{13}$, and a binary variable $d \in \{0, 1\}$ which indicates whether there is a far-away distal PAS in the MPRA sub-library. The model produces two outputs, a total proximal isoform proportion $\hat{y}_{\text {iso}} \in \mathbb {R}$, and 3′ cleavage distribution $\hat{\varvec{y}} \in \mathbb {R}^{186}$ ($\hat{y}_{186}$ corresponds to total isoform cleavage). When using the model for variant prediction, we set $l_{4} = 1$ and $d = 1$. The variant log odds ratio $\text {LOR}(\hat{y}^{(\text {wt})}, \hat{y}^{(\text {var})})$ is calculated as the average of the isoform and cleavage outputs: $\text {LOR}(\hat{y}^{(\text {wt})}, \hat{y}^{(\text {var})}) = \left( \text {LOR}(\hat{y}^{(\text {wt})}_{\text {iso}}, \hat{y}^{(\text {var})}_{\text {iso}}) + \text {LOR}(\sum \nolimits _{j=s}^{e} \hat{y}^{(\text {wt})}_{j}, \sum \nolimits _{j=s}^{e} \hat{y}^{(\text {var})}_{j}) \right) / 2$, where $s = 57$ and $e = 107$ for the MPRA of Bogard et al. [36] and the 3′ aQTLs of Mittleman et al. [21], and $s = 1$ and $e=185$ otherwise.

The trained model was downloaded from https://github.com/johli/aparent/tree/master/saved_models.

***DeeReCT-APA:*** [34] An LSTM-based model trained on mouse 3′-sequencing data. The model takes as input a tensor $\varvec{x} \in \{0, 1\}^{P \times 455 \times 4}$, where $\varvec{x}_{p} \in \{0, 1\}^{455 \times 4}$ denotes the *p*:th PAS in a given 3′ UTR. When using the model for SNV prediction, we only pass two input PASs ($P = 2$)—the sequence of the PAS containing the mutation and a fixed distal PAS that we never change. The distal PAS was chosen as a strong sequence from the training data. We could not use the distal PAS from the variant MPRA, since the model’s input window was larger than the plasmid reporter 3′ UTR. By passing either the wildtype or variant sequence as the proximal PAS, the model returns the predicted wildtype and variant isoform proportions $\hat{y}^{(\text {wt})}$ and $\hat{y}^{(\text {var})}$. Given these predictions, we calculate $\text {LOR}(\hat{y}^{(\text {wt})}, \hat{y}^{(\text {var})})$.

The model was re-trained using the code from https://github.com/lzx325/DeeReCT-APA-repo.

***DeepPASTA (Rel Iso):*** [33] An ensemble of CNNs trained on human 3′ sequencing data [13]. We used the “Tissue-specific, relatively dominant” models. These tissue-specific model instances take as input two 200-nt one-hot coded sequences $\varvec{x}^{(p)}, \varvec{x}^{(d)} \in \{0, 1\}^{200 \times 4}$ (proximal and distal PAS) and one-hot coded representations $\varvec{s}^{(p)}, \varvec{s}^{(d)} \{0, 1\}^{200 \times 7}$ of their most probable secondary structures. When using the models for SNV prediction, we use a fixed distal PAS that we never change. By passing either the wildtype or variant sequence as the proximal PAS, the model returns the predicted wildtype and variant isoform proportions $\hat{y}^{(\text {wt})}$ and $\hat{y}^{(\text {var})}$. Given these predictions, we calculate $\text {LOR}(\hat{y}^{(\text {wt})}, \hat{y}^{(\text {var})})$. This is repeated for each tissue-specific model and the average LOR is used as the final prediction.

The trained models were downloaded from https://www.cs.ucr.edu/~aaref001/DeepPASTA_site.html.

***DeepPASTA (Site Pred):*** [33] This CNN ensemble takes a single one-hot coded sequence $\varvec{x} \in \{0, 1\}^{200 \times 4}$ and one-hot coded representations $\varvec{s}^{(1)}, \varvec{s}^{(2)}, \varvec{s}^{(3)} \{0, 1\}^{200 \times 7}$ of the three most probable secondary structures, as input. The model predicts the likelihood of $\varvec{x}$ being a PAS. By passing either the wildtype or variant sequence as $\varvec{x}$, the model returns the predicted wildtype and variant PAS probabilities $\hat{y}^{(\text {wt})}$ and $\hat{y}^{(\text {var})}$. Given these predictions, we calculate $\text {LOR}(\hat{y}^{(\text {wt})}, \hat{y}^{(\text {var})})$.

The trained model was downloaded from https://www.cs.ucr.edu/~aaref001/DeepPASTA_site.html.

***PolyApredictor:*** [35] An RNA/DNA expression level CNN and a 3′-cleavage CNN (as two separate networks) trained on a plasmid reporter MPRA of 3′ UTRs (assayed in K562 cells). The models each take a one-hot coded sequence $\varvec{x} \in \{0, 1\}^{250}$ as input, which represents the 3′ UTR, and predicts either the total $\log \frac{RNA}{DNA}$ level $\hat{y} \in \mathbb {R}$ or the per-nucleotide $\log \frac{RNA}{DNA}$ levels $\hat{\varvec{y}} \in \mathbb {R}^{250}$ across all potential cleavage positions of the 3′ UTR. To use these models for variant prediction, we pass either the wildtype or variant sequence as $\varvec{x}$ and calculate the predicted LOR as $\text {LOR}(\hat{y}^{(\text {wt})}, \hat{y}^{(\text {var})}) = \hat{y}^{(\text {var})} - \hat{y}^{(\text {wt})}$.

The trained models were downloaded from https://github.com/segallab/PolyApredictors.

### Motif discovery

To generate a representative selection of RNA binding protein motifs within human polyadenylation signals, we used APARENT2 to predict the isoform logit $\text {log } \hat{y}_{\text {iso}} / (1 - \hat{y}_{\text {iso}})$ for 20,000 randomly sampled 3′ UTR PASs from PolyADB_V3 (where $\hat{y}_{\text {iso}} = \sum \nolimits _{k=77}^{127} \hat{y}_{k}$). The PASs were restricted from having more than 7 consecutive adenine bases, the signals had to be alternatively used in tissue-pooled measurements, and the CSE had to have a hamming distance of at most 2nt from the consensus AATAAA motif. We used DeepSHAP [84] to obtain attribution scores for each PAS (64 reference patterns), which were clustered into sequence logos using TF-MoDISco [56] (sliding window $= 8$, flank size $= 5$, max seqlets $=40,000$, FDR $= 0.05$, # mismatches $= 0$).

The TF-MoDISco software was installed from https://github.com/kundajelab/tfmodisco.

### Pairwise and multi-PAS modeling

All APA models were benchmarked on the pairwise APA prediction task using the four endogenous data sources from Muller et al. [57], Derti et al. [13], Lianoglou et al. [42], and Wang et al. [54]. For each dataset, we estimated the true isoform logit of each pair of APA sites as $\text {logit}_{\text {endogenous}} = \text {logit}\left( (c_{\text {p}} + c_{\text {pseudo}}) / (c_{\text {p}} + c_{\text {d}} + c_{\text {pseudo}}) \right)$, where $c_{\text {p}}$ and $c_{\text {d}}$ are the proximal and distal isoform counts and $c_{\text {pseudo}} = 0.5$ is a pseudo count. We then used each APA model to predict logit scores $\text {logit}_{\text {p}}$ and $\text {logit}_{\text {d}}$. These scores and the log-distance *d* between the sites were used to regress the measured isoform logits (Eq. 7):

7 $$\begin{aligned} \text {logit}_{\text {endogenous}} = w^{(\text {proximal})} \times \text {logit}_{\text {p}} + w^{(\text {distal})} \times \text {logit}_{\text {d}} + w^{(\text {distance})} \times d + w^{(\text {bias})} \end{aligned}$$

For the multi-PAS task, we estimated the distal isoform proportion of each gene as $y_{d} = c_{d} / \sum \nolimits _{i=1}^{10} c_{i}$. We then feed each APA model the 10 input PAS sequences of the gene (with zero padding if the gene has less than 10 PASs). Each model returns 10 predicted logit scores $\text {logit}_{i}$, which are used in a masked softmax regression model to predict the distal isoform proportion of the endogenous data (Eq. 8):

8 $$\begin{aligned} \hat{y}_{d} = \frac{ \text {exp} \left( f_{d} \right) }{ \sum \nolimits _{i=1}^{10} \mathbbm {1}_{\{\text {PAS } i \text { exists}\}} \times \left[ \text {exp} \left( f_{p, i} \right) \right] } \end{aligned}$$

where

$$\begin{aligned} f_{d}&= w_{d}^{(\text {score})} \times \text {logit}_{d} + \left[ \sum \limits _{k=1}^{4} w_{d, k}^{(\text {saluki})} \times \text {saluki}_{d, k} \right] + w_{d}^{(\text {distance})} \times d_{d} + w_{d}^{(\text {bias})}\\ f_{p, i}&= w_{p}^{(\text {score})} \times \text {logit}_{i} + \left[ \sum \limits _{k=1}^{4} w_{p, k}^{(\text {saluki})} \times \text {saluki}_{i, k} \right] + w_{p}^{(\text {distance})} \times d_{i} + w_{p}^{(\text {bias})} \end{aligned}$$

The variable $d_{i}$ denotes the cumulative log distance between PAS *i* and the proximal-most PAS. The variables $\left\{ \text {saluki}_{i, k}\right\} _{k=1}^{4}$ denote the first 4 principal components (PCs) of the final hidden-layer activations of the Saluki model [43] for isoform *i*. These 4 PCs explained ${>}95\%$ of the variance for all data sources. Since there are 50 training folds of the Saluki model, we train 50 corresponding APA regression models according to Eq. 8 and use the mean predicted distal isoform proportion in all evaluations. Saluki was downloaded from https://zenodo.org/record/6326409. Each 3′ UTR isoform was extracted from the GENCODE v19 annotation starting from the last defined stop codon of each gene and ended at the median cleavage site of the PAS [85]. A constant 5′ UTR and ORF, taken from https://github.com/vagarwal87/saluki_paper, were used for all 3′ UTRs. Note that the Saluki inputs were only used for the model named “A2+Saluki” in the benchmark of Fig. 1G. The parameters of the softmax regression model were fit using LM-BFGS. In one of the tests in Fig. 1G, the APARENT2 logits, the Saluki PCs, and the PAS log distances were used to fit a single-layer LSTM model with 16 hidden units instead of the (linear) softmax regression model. This model was trained in Keras with 20-fold cross-validation [81].

In Fig. 6A–D, we used the softmax regression model of Eq. 8 to score the total impact of 3′ aQTLs on pA efficiency and isoform stability. We trained the regression weights on tissue-pooled measurements from PolyADB V3 and relied on the annotations of this dataset to define the isoforms. The PolyADB annotation was used in favor of the APA annotation used to originally define the 3′ aQTLs because the latter annotation changes depending on the tissue type and even the specific variant. As it is difficult to train the model of Eq. 8 on such a dynamically changing annotation, we used the PolyADB annotation as a feasible approximation. However, as these annotation differences sometimes invert the sign of the prediction, we only used the absolute value of effect sizes for comparisons. We used isotonic regression in scikit-learn [86] to calibrate the predicted effect sizes on held-out SNPs that only occur in other tissue types than the tissue we are currently scoring.

### Tissue-specific modeling of native APA

We again used the tissue-specific RNA-seq data from Lianoglou et al. [42], but we now keep track of the total read count $c_{ij}^{(k)}$ supporting APA isoform *i* in tissue *j* of gene *k*. We removed genes with more than 10 APA isoforms. We estimated isoform proportions by normalizing the isoform counts by the total count across each gene: $y_{ij}^{(k)} = c_{ij}^{(k)} / \sum \nolimits _{t=1}^{10} c_{tj}^{(k)}$. We then created separately filtered copies of the data for pairs of tissues, where one tissue was HEK293 and the other tissue was either the testis, ovary, BLCL, or brain. Genes with less than 10 reads in any tissue were removed from each dataset. This resulted in 4453 HEK293–testis genes, 4495 HEK293–ovary genes, 4366 HEK293–BLCL genes, and 4715 HEK293–brain genes.

Using these data, we trained 4 individual tissue-specific models that learn the difference in isoform proportion $\Delta _{\text {HEK293}}^{\text {tissue}} = y_{i, \text {tissue}} - y_{i, \text {HEK293}}$ between the target tissue type and HEK293. The model works as follows: Given the 10 input PAS sequences $\varvec{x} \in \{0, 1\}^{10 \times 205 \times 4}$ of a given gene (with appropriate zero-padding), we execute APARENT2 on each PAS to obtain baseline cleavage predictions $\hat{\varvec{y}} \in [0, 1]^{10 \times 206}$. We compute the baseline isoform logit for PAS *i* as $\text {logit}_{i, \text {base}} = \text {logit} \left( \sum \nolimits _{j=1}^{205} \hat{y}_{ij} \right)$. We also feed $\varvec{x}$ as input to a trainable CNN that predicts tissue-specific scores $\hat{\varvec{s}} \in \mathbb {R}^{10 \times 2}$. The CNN weights are shared across all 10 PASs. Internally, the tissue-CNN consists of 2 convolutional layers (16 filters, 8 positions wide) and global average pooling. Finally, we linearly combine $\text {logit}_{i, \text {base}}$, $\hat{s}_{i, \text {tissue}}$ and the log distance $d_{i}$ between PAS *i* and the proximal-most PAS and apply masked softmax to predict tissue-specific proportions $\hat{y}_{i, \text {tissue}}$ (Eq. 9):

9 $$\begin{aligned} \hat{y}_{i, \text {tissue}} = \frac{ \mathbbm {1}_{\{\text {PAS } i \text { exists}\}} \times \left[ \text {exp} \left( f_{i} \right) \right] }{ \sum \nolimits _{t=1}^{10} \mathbbm {1}_{\{\text {PAS } t \text { exists}\}} \times \left[ \text {exp} \left( f_{t} \right) \right] } \end{aligned}$$

where

$$\begin{aligned} f_{i} = w_{i}^{(\text {score})} \times \left( \text {logit}_{i, \text {base}} + \hat{s}_{i, \text {tissue}} \right) + w_{i}^{(\text {distance})} \times d_{i} + w_{i}^{(\text {bias})} \end{aligned}$$

For the first 25 epochs, we froze the tissue-CNN weights and forced $\hat{s}_{i, \text {tissue}}$ to be 0. We only optimized the PAS-specific regression weights $\varvec{w} \in \mathbb {R}^{10 \times 3}$ from Eq. 9. We minimized the mean (masked) KL divergence between predicted and observed tissue-specific isoform proportions across all *K* genes (Eq. 10). Consequently, the regression weights $\varvec{w}$ will learn to combine the baseline APARENT2 scores and the log distances to infer the mean isoform proportion across the 2 tissues.

10 $$\begin{aligned} KL\left[ \hat{\varvec{y}} || \varvec{y} \right] = \frac{1}{K} \times \left( \sum \limits _{k=1}^{K} \sum \limits _{i=1}^{10} \mathbbm {1}_{\{\text {PAS } i \text { exists}\}} \times \left[ y_{i, \text {tissue}}^{k} \times \log \left( \frac{y_{i, \text {tissue}}^{k}}{\hat{y}_{i, \text {tissue}}^{k}}\right) + y_{i, \text {HEK293}}^{k} \times \log \left( \frac{y_{i, \text {HEK293}}^{k}}{\hat{y}_{i, \text {HEK293}}^{k}}\right) \right] \right) \end{aligned}$$

After the weights $\varvec{w}$ converge, we un-froze the tissue-CNN weights and optimized $\varvec{w}$ jointly with the CNN-predicted scores $\hat{s}_{i, \text {tissue}}$ for 25 additional epochs. We noticed that if we keep minimizing the KL divergence loss of Eq. 10, the CNN would disregard learning about tissue-specific differences (which is a relatively small source of variation for APA) in favor of learning to better predict the mean proportion across both tissues. We thus switched to a (masked) margin loss which penalized the model based on the observed and predicted tissue-specific differences $\Delta _{\text {HEK293}}^{\text {tissue}} = \left( y_{i, \text {tissue}} - y_{i, \text {HEK293}} \right)$ and $\hat{\Delta }_{\text {HEK293}}^{\text {tissue}} = \left( \hat{y}_{i, \text {tissue}} - \hat{y}_{i, \text {HEK293}} \right)$ rather than absolute proportions (Eq. 11).

11 $$\begin{aligned} \mathcal {L}\left[ \hat{\varvec{y}}, \varvec{y} \right] = \frac{1}{K} \times \left( \sum \limits _{k=1}^{K} \sum \limits _{i=1}^{10} \mathbbm {1}_{\{\text {PAS } i \text { exists}\}} \times \left[ \mathbbm {1}_{\{ |\Delta _{\text {HEK293}}^{\text {tissue}}| > 0.2 \}} \times \max \left( \text {sign}(\Delta _{\text {HEK293}}^{\text {tissue}}) \times (\Delta _{\text {HEK293}}^{\text {tissue}} - \hat{\Delta }_{\text {HEK293}}^{\text {tissue}}), 0 \right) \right] \right) \end{aligned}$$

For each tissue-specific model (testis, ovary, BLCL, brain), we learned an ensemble of 10 independently trained CNNs. We stopped when the validation error on a held-out test set of 500 genes started to increase.

### Tissue-specific aQTL effect size prediction

We used the testis-, ovary-, BLCL-, and brain-specific APA models to scale the effect size predictions made by APARENT2 on the GTEx aQTLs [20]. We used the linear model proposed by Cheng et al. [65] for combining baseline variant predictions with a tissue-specific scaling factor. Specifically, the logit of the tissue-specific distal isoform usage (PDUI) for the variant sequence is assumed to follow the relationship of Eq. 12.

12 $$\begin{aligned} \text {logit}\left( y_{\text {PDUI}, \text {tissue}}^{(var)} \right) = \text {logit}\left( y_{\text {PDUI}, \text {base}}^{(ref)} \right) + \beta _{\text {var}} + \beta _{\text {tissue}} + \beta _{\text {var} \times \text {tissue}} + \epsilon \end{aligned}$$

Here, $y_{\text {PDUI}, \text {base}}^{(ref)}$ corresponds to the mean PDUI measured across all tissues and samples. If we set

- $\beta _{\text {var}} = \lambda \times \text {LOR}(\hat{y}^{(\text {wt})}, \hat{y}^{(\text {var})})$ (the baseline APARENT2 variant prediction)
- $\beta _{\text {tissue}} = \gamma \times \left( \hat{s}_{\text {tissue}}^{(\text {ref})} - \hat{s}_{\text {HEK293}}^{(\text {ref})} \right)$ (the tissue-specific prediction)
- $\beta _{\text {var} \times \text {tissue}} = \gamma \times \left( \left( \hat{s}_{\text {tissue}}^{(\text {var})} - \hat{s}_{\text {HEK293}}^{(\text {var})} \right) - \left( \hat{s}_{\text {tissue}}^{(\text {ref})} - \hat{s}_{\text {HEK293}}^{(\text {ref})} \right) \right)$ (the tissue-specific variant effect)

and rearrange the terms, we get:

13 $$\begin{aligned} \Delta y_{\text {PDUI}, \text {tissue}}^{(var)} = \left[ \frac{1}{\left( 1 + e^{-\gamma \times \left( \hat{s}_{\text {tissue}}^{(\text {var})} - \hat{s}_{\text {HEK293}}^{(\text {var})} \right) } \times e^{-\lambda \times \text {LOR}(\hat{y}^{(\text {wt})}, \hat{y}^{(\text {var})})} \times \frac{1 - y_{\text {PDUI}, \text {base}}^{(ref)}}{y_{\text {PDUI}, \text {base}}^{(ref)}}\right. } - y_{\text {PDUI}, \text {base}}^{(ref)} \right] \end{aligned}$$

Compared to Eq. 6, the difference is that we scale the variant odds ratio $e^{-\lambda \times \text {LOR}(\hat{y}^{(\text {wt})}, \hat{y}^{(\text {var})})}$ predicted by APARENT2 with a tissue-specific odds ratio prediction $e^{-\gamma \times \left( \hat{s}_{\text {tissue}}^{(\text {var})} - \hat{s}_{\text {HEK293}}^{(\text {var})} \right) }$. Note that there is a free hyper-parameter $\gamma \in \mathbb {R}$ that we need to tune on the GTEx aQTL data in order to properly scale the score residual $\left( \hat{s}_{\text {tissue}}^{(\text {var})} - \hat{s}_{\text {HEK293}}^{(\text {var})} \right)$ predicted by the tissue model. We used the same value of $\gamma$ for all tissue types ($\gamma$ is chosen so as to maximize the median Spearman *r *against measured 3′ aQTLs for all tissues).

### Mask-based variant interpretation

We adapted our recent work on mask-based interpretation [49] to find the contextual features within a sequence that explain the *relative* fold change between wildtype and variant predictions. If the effect of all nucleotides were independent, the solution would simply be to return the mutated position itself (and nothing else). But, assuming mutations interfere with complex cis-regulatory code, the mask would have to retain a larger set of nucleotides (distant motifs, etc.) to reconstruct the variant effect. This is different from our earlier work, which focused on finding salient features that explain the *absolute* prediction of individual sequences. We found that per-example attribution worked stably for this task, so for simplicity, we settled on optimizing individual masks rather than training a parametric ad-hoc interpreter and fine-tuning its scores.

Let $\varvec{s} \in (0, +\infty ]^{N}$ be the scores (the “mask”) that we will optimize specifically for the wildtype and variant sequences $\varvec{x}^{(\text {wt})}$ and $\varvec{x}^{(\text {var})}$ of length *N*. We first set $s_{\text {u}} = +\infty$ and freeze this score, where *u* is the position of the mutation. Next, we channel-broadcast $\varvec{s}$ into $\dot{\varvec{s}} \in (0, +\infty ]^{N \times 4}$ (same shape as $\varvec{x}^{(\text {wt})}$):

14 $$\begin{aligned} \dot{s_{ij}} = s_{i} \quad (1 \le i \le N, 1 \le j \le 4) \end{aligned}$$

We then use $\dot{\varvec{s}}$ as interpolation coefficients between the original wildtype pattern $\varvec{x}^{(\text {wt})}$ and a reference pattern $\tilde{\varvec{b}}(\varvec{x}^{(\text {wt})})$, which is taken here as a Laplace-smoothed copy of the wildtype pattern; $\tilde{\varvec{b}}(\varvec{x}^{(\text {wt})})_{ij} = \left( \varvec{x}^{(\text {wt})}_{ij} + 1\right) / 5$:

15 $$\begin{aligned} \hat{\varvec{x}}_{\varvec{s}}^{(\text {wt})} = \sigma \left( \log \tilde{\varvec{b}}(\varvec{x}^{(\text {wt})}) + \varvec{x}^{(\text {wt})} \times \dot{\varvec{s}} \right) \end{aligned}$$

Here, $\sigma$ denotes position-wise softmax, i.e., $\hat{\varvec{x}}_{\varvec{s}}^{(\text {wt})}$ is a softmax-relaxed position-specific scoring matrix (PSSM) whose entropy is controlled by $\varvec{s}$. Next, we sample a discrete one-hot coded pattern $\varvec{x}_{\varvec{s}}^{(\text {wt})}$ from $\hat{\varvec{x}}_{\varvec{s}}^{(\text {wt})}$ using the Gumbel distribution [87]:

16 $$\begin{aligned} \varvec{x}_{\varvec{s}}^{(\text {wt})} = \{ \mathbbm {C}_{i}^{(\text {wt})} \}_{i=1}^{N}, \quad \mathbbm {C}_{i}^{(\text {wt})} \sim \text {Gumbel}(\hat{\varvec{x}}_{\varvec{s}i1}^{(\text {wt})}, ..., \hat{\varvec{x}}_{\varvec{s}iM}^{(\text {wt})}) \end{aligned}$$

The next step is to construct a similar sample $\varvec{x}_{\varvec{s}}^{(\text {var})}$ of the variant pattern, whose information content has been masked and only the salient features marked by $\varvec{s}$ are conserved. While we could theoretically re-apply Eqs. 15-16 to $\varvec{x}_{\varvec{s}}^{(\text {var})}$ the same way we obtained $\varvec{x}_{\varvec{s}}^{(\text {wt})}$, that approach does not work well in practice. The reason is that if the wildtype and variant PSSMs $\hat{\varvec{x}}_{\varvec{s}}^{(\text {wt})}$ and $\hat{\varvec{x}}_{\varvec{s}}^{(\text {var})}$ have high entropy (which they are optimized for), then drawing independent samples from each PSSM will result in patterns with very different sequence content (except for the small set of features retained by $\varvec{s}$). Consequently, the variance in the resulting predictions will be unnecessarily high. Instead, we directly construct the mutated sample $\varvec{x}_{\varvec{s}}^{(\text {var})}$ from the wildtype sample $\varvec{x}_{\varvec{s}}^{(\text {wt})}$ by “erasing” the wildtype nucleotide and adding the mutation:

17 $$\begin{aligned} \varvec{x}_{\varvec{s}}^{(\text {var})} = \varvec{x}_{\varvec{s}}^{(\text {wt})} + \left( \varvec{x}^{(\text {var})} - \varvec{x}^{(\text {wt})} \right) \end{aligned}$$

Both samples $\varvec{x}_{\varvec{s}}^{(\text {var})}$ and $\varvec{x}_{\varvec{s}}^{(\text {wt})}$ now have the same randomized (masked) background content and the same feature set retained by $\varvec{s}$. Finally, we optimize the cost defined in Eq. 18, which minimizes the mean squared error between the original and scrambled log odds ratio-predictions while maximizing entropy.

18 $$\begin{aligned} \min _{\varvec{s}} \, \left( \Delta _{\varvec{s}} - \Delta \right) ^{2} + \lambda \cdot \frac{1}{N} \cdot \text {KL}\left[ \tilde{\varvec{b}}(\varvec{x}^{(\text {wt})}) || \hat{\varvec{x}}_{\varvec{s}}^{(\text {wt})} \right] \end{aligned}$$

Here, $\Delta _{\varvec{s}} = \text {LOR}\left( \mathcal {P}(\varvec{x}_{\varvec{s}}^{(\text {wt})}), \mathcal {P}(\varvec{x}_{\varvec{s}}^{(\text {var})}) \right)$ and $\Delta = \text {LOR}\left( \mathcal {P}(\varvec{x}^{(\text {wt})}), \mathcal {P}(\varvec{x}^{(\text {var})}) \right)$, where $\mathcal {P}(\varvec{x})$ is the proximal isoform proportion predicted by APARENT2 ($\mathcal {P}(\varvec{x}) = \sum \nolimits _{j=77}^{127} y_{j}(\varvec{x})$ for predicted cleavage $\varvec{y}(\varvec{x})$). See Eq. 5 for a definition of LOR. Note that we do not optimize $\varvec{s}$ directly; instead, we optimize parameters $\varvec{w} \in \mathbb {R}^{N}$, which are instance-normalized and softplus-transformed into $\varvec{s}$ ($\varvec{s} = \text {Softplus}(\text {IN}(\varvec{w}))$). In our experiments, we optimize $\varvec{w}$ for 300 iterations of gradient descent (Adam, learning rate $= 0.01$). We noticed more stable performance if we first optimize the mask $\varvec{s}$ for a small target KL divergence $t_{\text {bits}}$ (Eq. 19) for the first few gradient updates before maximizing the entropy unbounded.

19 $$\begin{aligned} \min _{\varvec{s}} \, \left( \Delta _{\varvec{s}} - \Delta \right) ^{2} + \lambda \cdot \left( \frac{1}{N} \cdot \text {KL}\left[ \tilde{\varvec{b}}(\varvec{x}^{(\text {wt})}) || \hat{\varvec{x}}_{\varvec{s}}^{(\text {wt})} \right] - t_{\text {bits}} \right) ^{2} \end{aligned}$$

### ASD cohort data filtering procedure

The autism spectrum disorder (ASD) WGS data from An et al. [51] was filtered by different criteria in some of the figures. In Additional file 1: Fig. S7C (left), we remove variants (in cases and controls) that occur in a PAS which shares common variants in gnomAD (AF ${>}0.01\%$) with strictly larger effect sizes or with ${>}$1.5-fold effect sizes. In Additional file 1: Fig. S7C (right), we apply more stringent filtering (1.25-fold effect size cutoff) and we also remove variants that occur in PASs with a downstream neighboring PAS within 200nt in PolyADB v3. In main Fig. 7G, we re-processed the gnomAD data by binning SNVs in the same PAS by their effect size (5 bins) and recomputed their (joint) allele frequency by aggregating allele counts in each bin. This allows for a group of rare variants (in the same PAS) to take the role of one common variant if their effect sizes are comparable. The same filtering procedure was used for the cohort data from Yuen et al. [78] (Additional file 1: Fig. S7D), but the gnomAD AF cutoff was raised to $0.1\%$ due to the smaller sample size. For both datasets, whenever a variant overlaps multiple PASs, we assign the mutation to the PAS with largest predicted effect size (we tested other assignment strategies in Additional file 1: Fig. S7E).

### MPRA validation of selected variants

A total of 76 predicted outlier variants from the filtered autism data were chosen for experimental validation (38 case variants and 38 matched controls from Additional file 1: Fig. S7C). Additionally, we tested 9 GWAS SNPs, 3 hand-picked examples from the F2, SCAF8, and MECP2 genes, 2 apaQTLs, 4 matched control SNPs from gnomAD, and 6 control PASs that had previously been measured in the MPRA of Bogard et al. [36].

### Cloning

For the variant and reference libraries, a vector was constructed by cloning two separate 250-nt oligo pool libraries (Twist) 25nt upstream of the bGH pA signal in an mCherry reporter plasmid. All libraries contained homology to the vector and were constructed using In-Fusion assembly (Takara). Library sizes were estimated by plating serial dilutions of library transformation and extrapolating plasmid coverage based on colony counts. The remaining transformants were grown in 100-mL LB overnight culture and libraries were prepared using a HiSpeed Plasmid Midi Kit (Qiagen). Selected individual clones from each library were Sanger sequenced to confirm library assembly. Library sequences and primers are listed in Additional file 2: Table S1.

### Cell culture and transfection

HEK 293T cells (ATCC, CRL-3216) were cultured in Dulbecco’s modified Eagle medium (Gibco), SK-N-SH cells (ATCC HTB-11) were cultured in MEM (Gibco), and HCM3 cells (ATCC, CRL-3304) were cultured in EMEM (ATCC), all supplemented with 10% fetal bovine serum (Cytiva) and 1% penicillin/streptomycin (Gibco). For transfections, 300,000 (HEK293T) or 500,000 (SK-N-SH, HMC3) cells were plated (2mL) in tissue culture-treated 6-well plates (Fisher Scientific) 24 h prior such that they would be 50–70% confluent on the day of transfection. Variant and reference libraries were transfected into cells using Lipofectamine 3000 (Thermo Fisher) with 2 biological replicates for each library. Media were changed 5 h post-transfection.

### RNA extraction

Cells were harvested for RNA extraction 36–48 h after transfection. Cells were detached by incubating 2–5 min at room temperature with 1mL of TrypLE (Gibco). Once cells were detached, they were added to 3mL of media with 10% FBS. Cells were rinsed twice with 1x PBS (Gibco). One to 5% of the cells were used for flow cytometry to confirm transfection efficiency and the remaining cells were lysed and passed through a QIAshredder (Qiagen) to homogenize cell lysates. Total RNA was extracted from lysates using the RNeasy kit (Qiagen) with additional on column DNaseI digestion performed following the manufacturer’s protocol. mRNA was isolated using the NEBNext Poly(A) mRNA Magnetic Isolation Module (NEB) with 5$\upmu$g of total RNA input per sample.

### Sequencing library construction

The resulting mRNAs were reverse transcribed with SuperScript IV Reverse Transcriptase (Thermo Fisher) to generate cDNA. Polyadenylated mRNA was reverse transcribed with an anchored polyT primer containing Illumina adaptor sequences, a unique molecular identifier(UMI), and unique index sequences for each sample (P7-index-PE2-UMI-T18VN). RNA hydrolysis was performed on cDNA and samples were purified using DNA Clean & Concentrator-5 (Zymo). Library cDNA was then amplified with KAPA HiFi HotStart ReadyMix (Roche) using a library-specific forward primer containing additional Illumina adaptor sequences (P5-PE1) and reverse primer matching the adaptor sequence added during RT. Amplification was conducted and monitored with qPCR and stopped early to minimize PCR biases. Samples were size selected to keep fragments $\ge$ 100nt using KAPA Pure Beads (Roche). Sample size distributions were analyzed using Tapestation High Sensitivity D1000 (Agilent).

### Library sequencing

Library concentrations were quantified using qPCR quantification with the NEBNext Library Quant Kit for Illumina (NEB) as well as Qubit 1x dsDNA HS (Thermo Fisher). Concentrations between the two methods were averaged to determine optimal library loading concentrations for sequencing. Libraries were pooled with 10% PhiX and sequenced on MiSeq (Illumina) with a MiSeq Reagent Nano or Micro v2 (300 cycles) kit. Paired-end sequencing was performed with read 1 (292 nt) covering the library sequences, read 2 (8 nt) covering the UMIs, and the index read (6 nt) for demultiplexing pooled samples.

### Barcoding and mapping

The open-source adaptor trimming software package cutadapt v1.15 was used to trim adapters off read 1. Read 1 was then aligned and mapped to the known respective library sequences. The sequence upstream of the proximal PAS CSE was used to map cleaved mRNA back to the full UTR sequence (allowing for $\le 2$ substitution errors). Reads were searched 5′ to 3′ across for the site of polyadenylation as sequencing reads were long enough to precisely locate cut sites for all proximal isoforms. The site of polyadenylation was identified by searching for a consecutive run of 20 A’s (allowing for $\le 2$ substitution errors). A read was considered distally cleaved if no polyadenylation site was found in read 1. Mapped reads were collapsed over UMIs in read 2. The final counts were pooled across the 2 replicates.

## Supplementary Information

Additional file 1. Supplementary Information.

Additional file 2. Supplementary Tables. Supplementary Table S1 (sequences and primers used in experiments).

Additional file 3. Review history.

## Data Availability

The MPRA data of Bogard et al. is available at https://github.com/johli/aparent [88]. The MPRA data of Slutskin et al. is available at https://github.com/segallab/PolyApredictors [89]. The APADB data is available at http://tools.genxpro.net:9000/apadb/download/track/hg19.apadb_v2_final.bed [90]. The RNA-seq data of Lianoglou et al. is available at https://cbio.mskcc.org/leslielab/ApA/atlas/ [91]. The RNA-seq data of Derti et al. is available on Gene Expression Omnibus (GSE30198) [92]. The PolyADB V3 data is available at https://exon.apps.wistar.org/PolyA_DB/v3/download/3.2/human_pas.zip [93]. The GTEx V7 3′ aQTL data is available at https://doi.org/10.7303/syn22236281 [94]. The GTEx V8 3′ aQTL data is available at https://wlcb.oit.uci.edu/3aQTLatlas [95]. The aQTL data of Mittleman et al. is available on Gene Expression Omnibus (GSE138197) [96]. CAUSALdb is available at http://www.mulinlab.org/causaldb/index.html [97]. The UK Biobank fine-mapping results are available at https://www.finucanelab.org/data [98]. The processed autism WGS data is available in Supplementary Table S2 of An et al. [51] and Supplementary Table S10 of Yuen et al. [78]. The MPRA data generated in this study is available on Gene Expression Omnibus (GSE214825) [99]. APARENT2 is available under an MIT License on GitHub at http://www.github.com/johli/aparent-resnet and Zenodo [100]. The variant prediction model is available online as an interactive web tool at https://apa.cs.washington.edu/. External software is listed in Section 5.
