# Supplementary material for: Deciphering the impact of genetic variation on human polyadenylation using APARENT2
Source: Genome Biol. 2022 Nov 5;23:232. doi: 10.1186/s13059-022-02799-4 (PMC9636789; doi:10.1186/s13059-022-02799-4)
Supplement: Supplementary file 1 — Additional file 1. Supplementary Information. [file 13059_2022_2799_MOESM1_ESM.pdf]

Supplementary Information for Paper: "Deciphering the Impact of Genetic Variation on Human Polyadenylation using APARENT2"

Johannes Linder[1*] , Samantha E. Koplik[2] , Anshul Kundaje[1,3] and Georg Seelig[4,5]

[1] Department of Genetics, Stanford University, Stanford, USA
[2] Department of Bioengineering, University of Washington, Seattle, USA
[3] Department of Computer Science, Stanford University, Stanford, USA
[4] Paul G. Allen School of Computer Science & Engineering, University of Washington, Seattle, USA
[5] Department of Electrical & Computer Engineering, University of Washington, Seattle, USA
[*] Correspondence: jlinder2@stanford.edu

Supplementary Figures

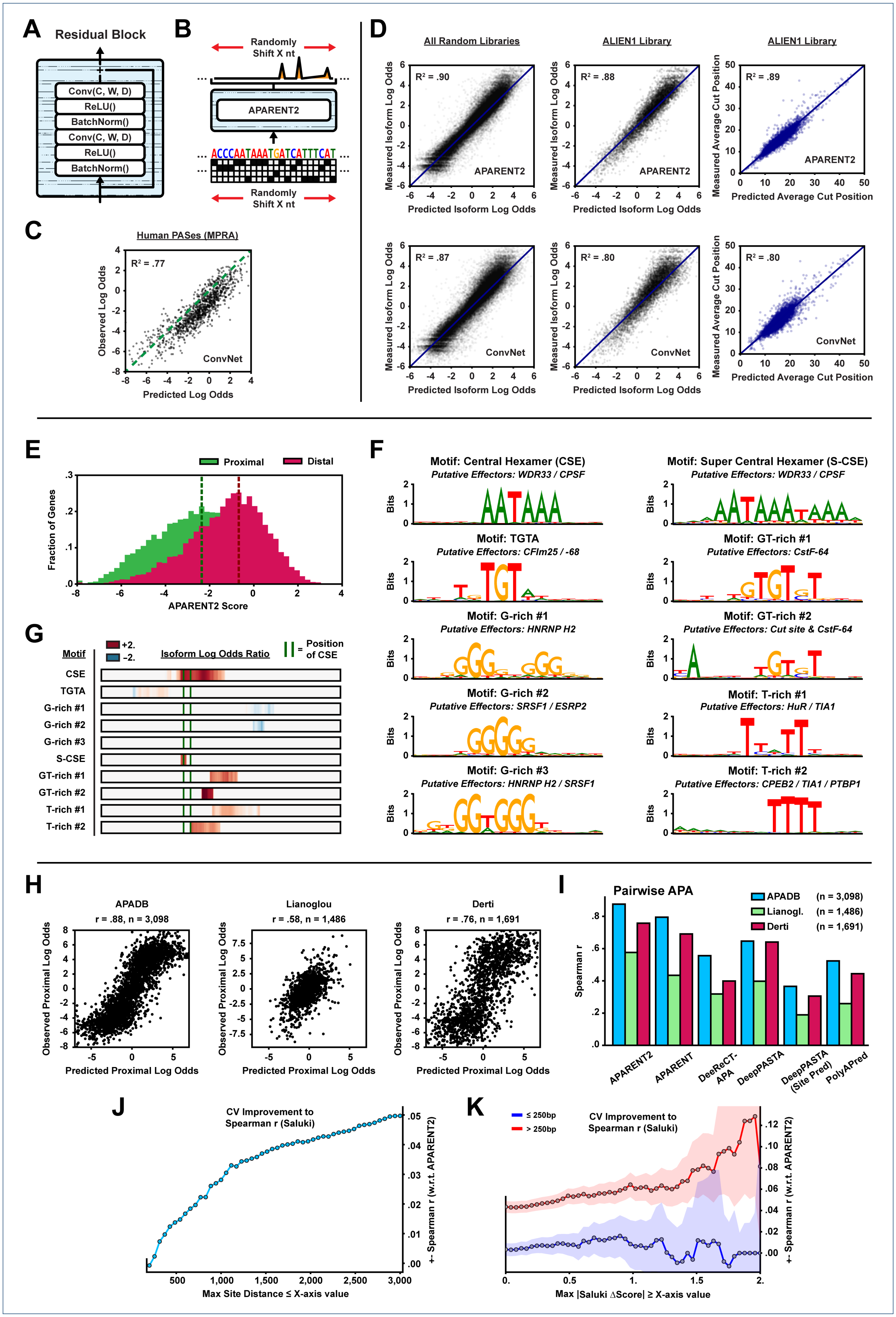
A
Residual Block
Conv(C, W, D)
ReLU()
BatchNorm()
Conv(C, W, D)
ReLU()
BatchNorm()
B
Randomly Shift X nt
APARENT2
ACCCAATAAATGATCATTTCAT
Randomly Shift X nt
C
Human PASes (MPRA)
R² = .77
ConvNet
Observed Log Odds
Predicted Log Odds
D
All Random Libraries
ALIEN1 Library
ALIEN1 Library
R² = .90
R² = .88
R² = .89
APARENT2
R² = .87
R² = .80
R² = .80
ConvNet
Measured Isoform Log Odds
Predicted Isoform Log Odds
Measured Average Cut Position
Predicted Average Cut Position
E
Proximal
Distal
Fraction of Genes
APARENT2 Score
F
Motif: Central Hexamer (CSE)
Putative Effectors: WDR33 / CPSF
Motif: Super Central Hexamer (S-CSE)
Putative Effectors: WDR33 / CPSF
Motif: TGTA
Putative Effectors: CFIm25 / -68
Motif: GT-rich #1
Putative Effectors: CstF-64
Motif: G-rich #1
Putative Effectors: HNRNP H2
Motif: GT-rich #2
Putative Effectors: Cut site & CstF-64
Motif: G-rich #2
Putative Effectors: SRSF1 / ESRP2
Motif: T-rich #1
Putative Effectors: HuR / TIA1
Motif: G-rich #3
Putative Effectors: HNRNP H2 / SRSF1
Motif: T-rich #2
Putative Effectors: CPEB2 / TIA1 / PTBP1
Bits
G
Motif
+2.
-2.
Isoform Log Odds Ratio
= Position of CSE
CSE
TGTA
G-rich #1
G-rich #2
G-rich #3
S-CSE
GT-rich #1
GT-rich #2
T-rich #1
T-rich #2
H
APADB
r = .88, n = 3,098
Lianoglou
r = .58, n = 1,486
Derti
r = .76, n = 1,691
Observed Proximal Log Odds
Predicted Proximal Log Odds
I
Pairwise APA
APADB (n = 3,098)
Lianogl. (n = 1,486)
Derti (n = 1,691)
Spearman r
APARENT2
APARENT
DeeReCT-APA
DeepPASTA
DeepPASTA (Site Pred)
PolyApred
J
CV Improvement to Spearman r (Saluki)
+- Spearman r (w.r.t. APARENT2)
Max Site Distance ≤ X-axis value
K
≤ 250bp
> 250bp
CV Improvement to Spearman r (Saluki)
+- Spearman r (w.r.t. APARENT2)
Max |Saluki ΔScore| ≥ X-axis value

Figure S1: **A** The internal architecture of a residual block. C = # of channels, W = filter width, D = dilation rate. **B** During training, the input sequences and their target distributions are randomly shifted by a number of nucleotides. **C** Predicted vs measured isoform log odds of held-out human PASs measured in the MPRA of Bogard et al. (2019) for the baseline ConvNet model ($n = 1,085$). **D** Predicted vs measured proximal isoform log odds of held-out test sequences from all MPRA libraries (left) ($n = 60,198$) or the ALIEN1 library only (middle) ($n = 7,755$), and predicted vs measured average cut position downstream of the CSE of ALIEN1 test sequences (right) ($n = 6,203$). **E** Distribution of predicted isoform log odds using APARENT2 for the proximal-most and distal-most PASs in the 3' UTR of $n = 12,503$ genes (PolyADB V3). **F** Selection of RNA binding protein (RBP) motifs generated by TF-MoDISco. Each motif is represented as a position weight matrix (PWM). The motifs were generated from the SHAP scores of $n = 20,000$ randomly sampled PAS sequences from PolyADB V3. **G** Estimated isoform log odds ratios in the presence of a given motif at a specific position in the sequence (i.e. the increase, *or* decrease, in predicted isoform log odds when a given motif is present). **H** Predicted vs observed isoform log odds between pairs of adjacent human 3' UTR PASs, as measured in three separate native transcriptomic datasets. Predictions are made by linear regression of the APARENT2 scores of the proximal and distal signals and their log-distance as features. Read counts were pooled across tissues. A minimum read count of 500 was used as cutoff for all three data sources. **I** Comparison of correlation between predicted and measured isoform log odds for pairs of adjacent human 3' UTR PASs. Each model predicts logit scores which are used to fit a pairwise APA regressor (20-fold cross-validation). **J** Improvement in spearman r when using Saluki scores in addition to APARENT2 as input, as a function of the maximum distance between any adjacent pair of PASs in a given gene (data source = APADB). **K** Improvement in spearman r as a function of the minimum difference in Saluki score between at least one pair of PASs (blue / red = genes with PAS distances $\leq$ 250bp / $>$ 250bp; shaded area = 90% confidence interval estimated by $10,000$-fold bootstrapping; median improvement across data sources).

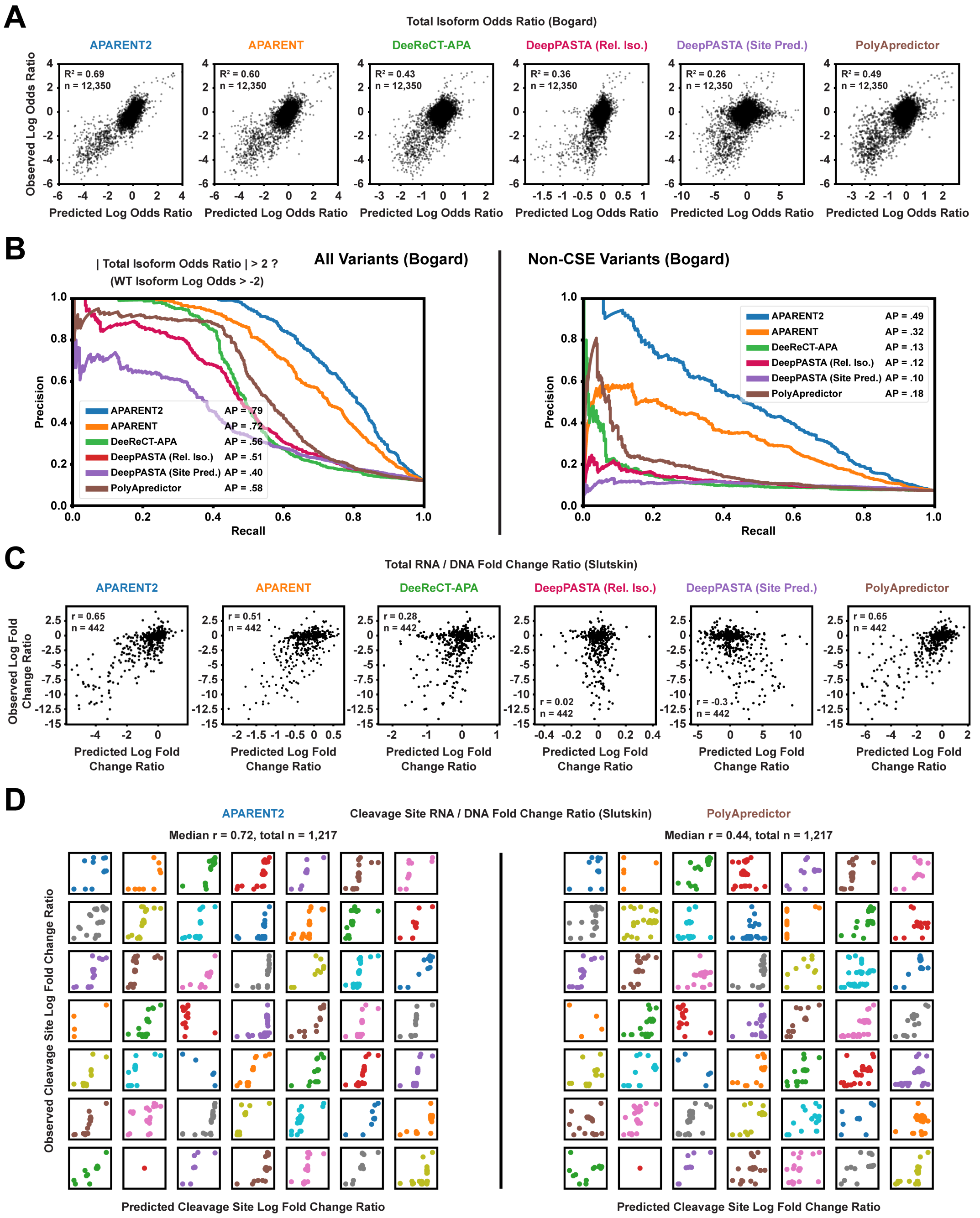

Figure S2: **A** Comparison of predicted vs measured variant isoform log odds ratios on the MPRA data from Bogard et al. (2019) ($n = 12,350$). Individual scatter plots are shown for each tested model. **B** Comparison of precision-recall curves when tasking each model with classifying disruptive APA variants on the data from Bogard et al. (2019). Left: All variants. Right: Non-CSE variants only. The data only includes variants with a wildtype isoform log odds > -2. **C** Comparison of predicted vs measured RNA/DNA log fold change ratios on the data from Slutskin et al. (2019). Individual scatter plots are shown for each tested model. Only variants from the PolyApredictor test set are included ($n = 442$). **D** Comparison of predicted vs measured cleavage site RNA/DNA log fold change ratios on the scanning mutagenesis data from Slutskin et al. (2019). Individual scatter plots are shown for each wildtype UTR. Each data point corresponds to one cleavage position. Total $n = 1,217$.

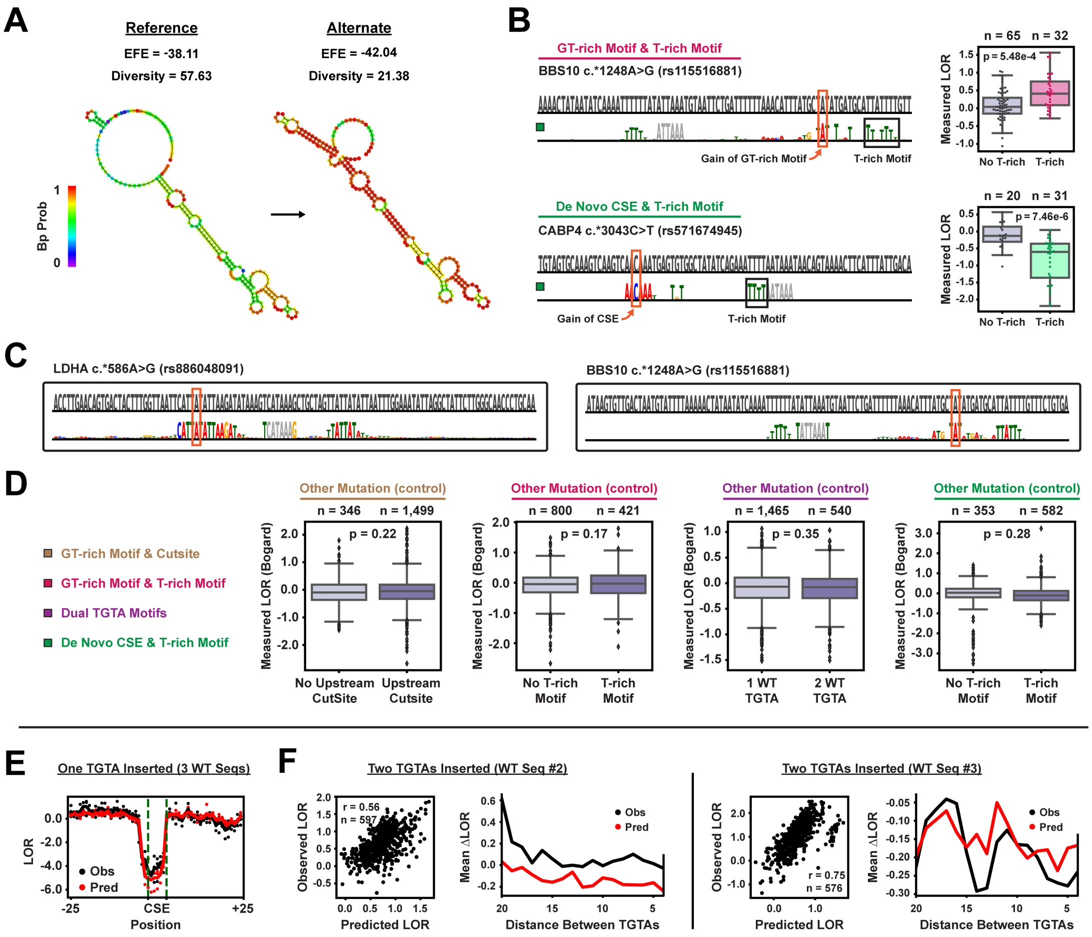

Figure S3: **A** Secondary structure (thermodynamic free energy ensemble) of the wildtype and mutated PAS for variant rs886048091. Plots are produced with Vienna RNAFold. EFE = Ensemble free energy, Diversity = Ensemble diversity. **B** Mask-based interpretation of two ClinVar SNVs: (1) a variant that creates a GT-rich motif in the DSE (rs115516881) and (2) a variant that creates a de novo central hexamer element in the USE of an existing PAS (rs571674945). Both attributions highlight distal T-rich motifs as necessary determinants for reconstructing the predicted log odds ratios (LORs). The boxplots show measured LORs from the MPRA of Bogard et al. (2019). The p-values are computed with a two-sided t-test. **C** Mask-based interpretation of example variants rs886048091 and rs115516881 with a fixed Gaussian filter at the final scrambling layer (3 positions wide). **D** Control tests related to main Fig. 3C and supplementary Fig. S3B. Each boxplot shows the measured LOR of arbitrary variants measured in the MPRA, separated into two categories based on whether the proposed epistatic motif is present in the wildtype sequence or not. The p-values are computed with a two-sided t-test. **E** Individual TGTA motifs are inserted at every possible position in 3 wildtype PASs. The plot shows predicted and measured LOR as a function of insert position relative to the CSE. **F** Two TGTA motifs are inserted at every possible motif distance in the USE of two wildtype PASs. The plots show predicted vs measured LOR of each insertion and average epistasis (the difference in LOR between the dual insertion and the sum of each individual insertion) as a function of motif distance.

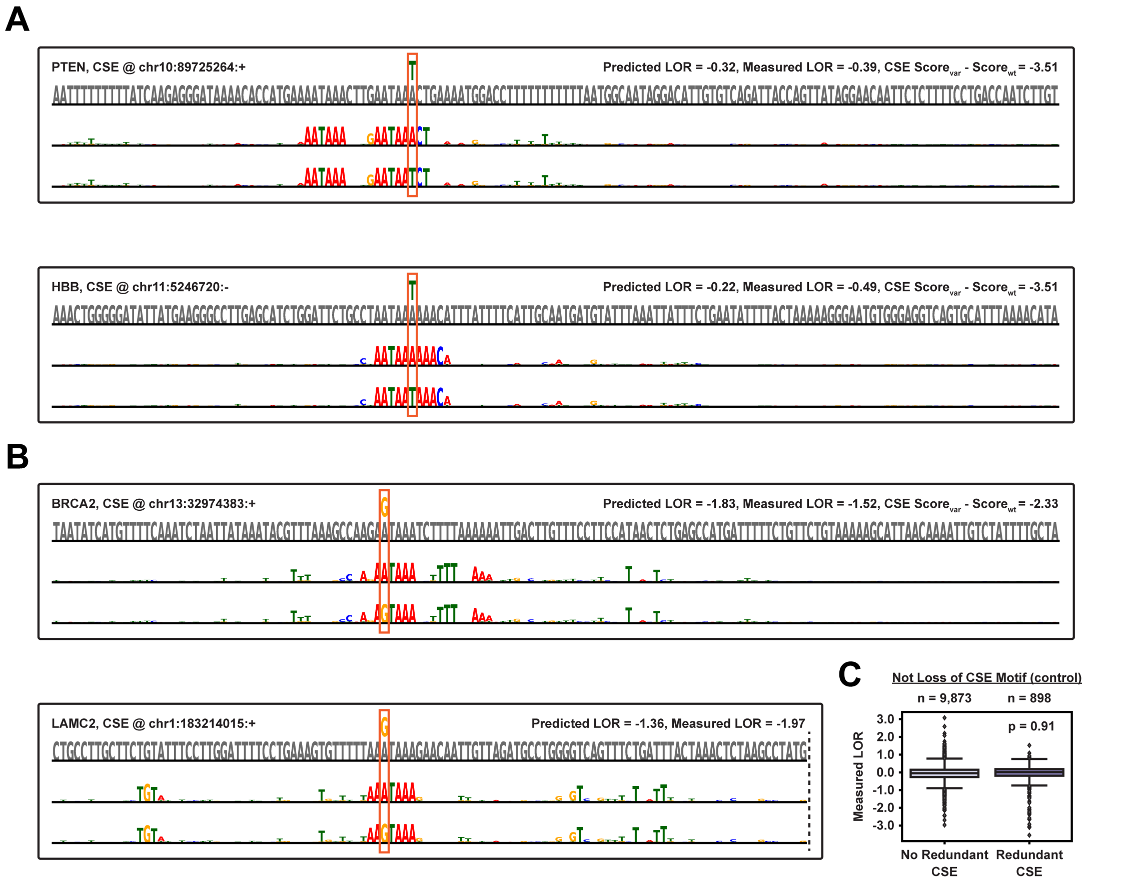

Figure S4: **A** Additional mask-based interpretations of functionally silent CSE mutations in the PTEN- and HBB genes. Annotated on the right are predicted and observed isoform log odds ratios (as measured in an MPRA), as well as CSE hexamer regression scores. **B** Additional interpretations of variants with dampened effect sizes (with respect to a linear hexamer regression model) in the BRCA2- and LAMC2 genes. Predicted and measured log odds ratios on the right. **C** Boxplot showing measured log odds ratios (LORs) from the MPRA of Bogard et al. (2019), for arbitrary mutations that occur in PASs with a single CSE hexamer or in PASs with additional redundant CSE hexamers. The p-value is computed with a two-sided t-test.

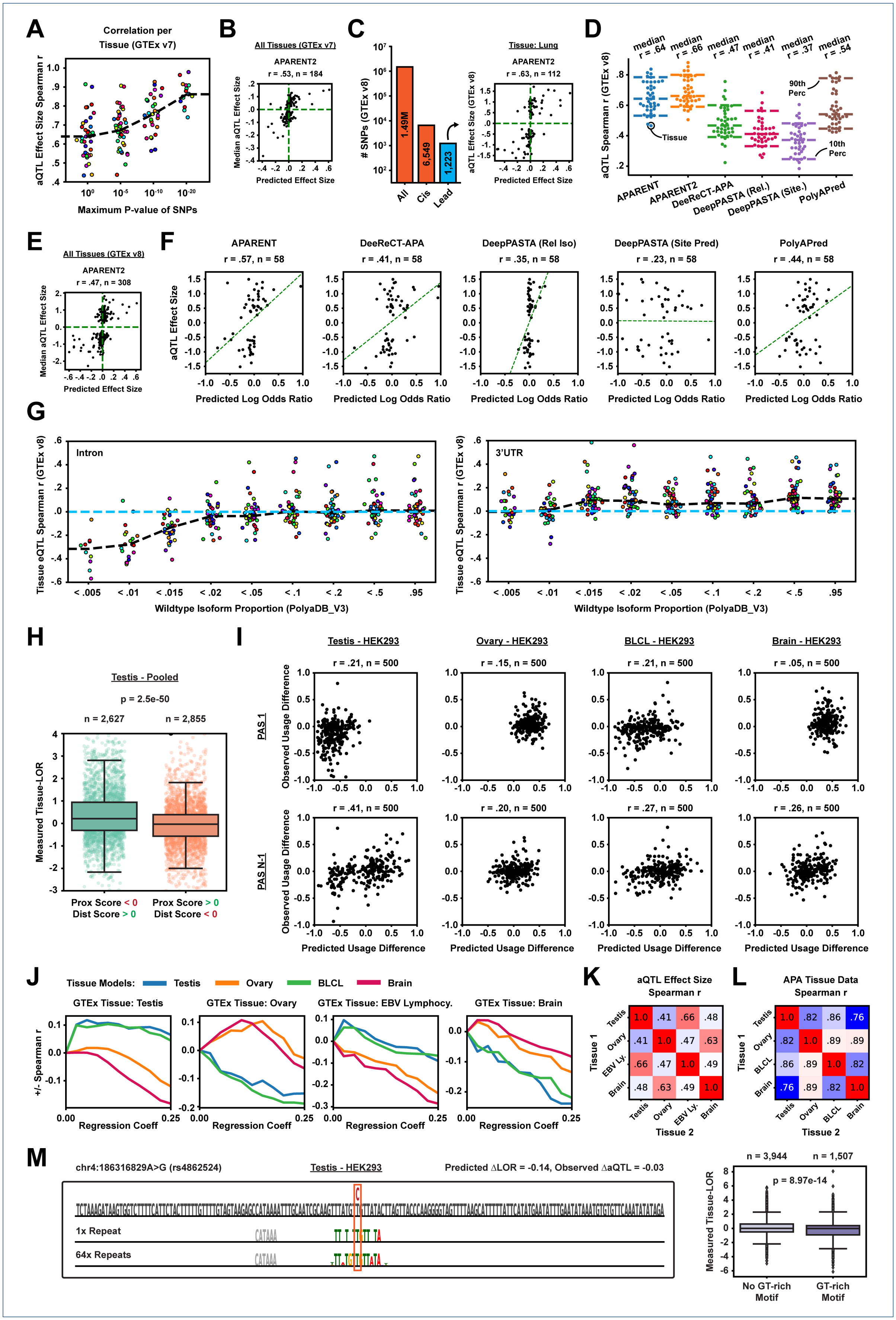
A
Correlation per Tissue (GTEx v7)
aQTL Effect Size Spearman r
Maximum P-value of SNPs
B
All Tissues (GTEx v7)
APARENT2
r = .53, n = 184
Median aQTL Effect Size
Predicted Effect Size
C
SNPs (GTEx v8)
1.49M
6,549
1,223
All
Cis
Lead
Tissue: Lung
APARENT2
r = .63, n = 112
aQTL Effect Size (GTEx v8)
Predicted Effect Size
D
median r = .64
median r = .66
median r = .47
median r = .41
median r = .37
median r = .54
aQTL Spearman r (GTEx v8)
90th Perc
10th Perc
Tissue
APARENT
APARENT2
DeeReCT-APA
DeepPASTA (Rel.)
DeepPASTA (Site)
PolyAPred
E
All Tissues (GTEx v8)
APARENT2
r = .47, n = 308
Median aQTL Effect Size
Predicted Effect Size
F
APARENT r = .57, n = 58
DeeReCT-APA r = .41, n = 58
DeepPASTA (Rel Iso) r = .35, n = 58
DeepPASTA (Site Pred) r = .23, n = 58
PolyAPred r = .44, n = 58
aQTL Effect Size
Predicted Log Odds Ratio
G
Intron
3'UTR
Tissue eQTL Spearman r (GTEx v8)
Wildtype Isoform Proportion (PolyaDB_V3)
H
Testis - Pooled
p = 2.5e-50
n = 2,627
n = 2,855
Measured Tissue-LOR
Prox Score < 0
Dist Score > 0
Prox Score > 0
Dist Score < 0
I
Testis - HEK293
Ovary - HEK293
BLCL - HEK293
Brain - HEK293
PAS 1
PAS N-1
r = .21, n = 500
r = .15, n = 500
r = .21, n = 500
r = .05, n = 500
r = .41, n = 500
r = .20, n = 500
r = .27, n = 500
r = .26, n = 500
Observed Usage Difference
Predicted Usage Difference
J
Tissue Models: Testis Ovary BLCL Brain
GTEx Tissue: Testis
GTEx Tissue: Ovary
GTEx Tissue: EBV Lymphocy.
GTEx Tissue: Brain
+/- Spearman r
Regression Coeff
K
aQTL Effect Size Spearman r
Tissue 1
Tissue 2
Testis Ovary EBV Ly. Brain
1.0 .41 .66 .48
.41 1.0 .47 .63
.66 .47 1.0 .49
.48 .63 .49 1.0
L
APA Tissue Data Spearman r
Tissue 1
Tissue 2
Testis Ovary BLCL Brain
1.0 .82 .86 .76
.82 1.0 .89 .89
.86 .89 1.0 .82
.76 .89 .82 1.0
M
chr4:186316829A>G (rs4862524)
Testis - HEK293
Predicted ΔLOR = -0.14, Observed ΔaQTL = -0.03
1x Repeat
64x Repeats
n = 3,944
n = 1,507
p = 8.97e-14
Measured Tissue-LOR
No GT-rich Motif
GT-rich Motif

Figure S5: **A** Correlation between predicted isoform log odds ratios (LORs; using APARENT2) and estimated 3' aQTL effect sizes from the GTEx v7 atlas. Each dot corresponds to the spearman r correlation for a particular tissue, after having filtered the set of SNPs to those with a p-value below the cutoff specified by the x-axis. Number of unique lead cis-aQTLs across all tissues = 366. **B** Predicted effect sizes (using APARENT2) vs median 3' aQTL effect sizes taken across all tissues of the GTEx v7 atlas. Only lead SNPs with support in more than 1 tissue are included. **C** Left: Number of 3' aQTLs, cis-acting 3' aQTLs and lead 3' aQTLs respectively in the newer GTEx v8 atlas. Right: Predicted vs measured 3' aQTL effect sizes in Lung (GTEx v8). **D** Predicted vs measured 3' aQTL effect size spearman r's (GTEx v8). Each dot corresponds to the spearman r for a given APA model in a particular tissue type. The dashed horizontal lines denote the 10th, 50th and 90th percentiles of each distribution. **E** Predicted effect sizes (using APARENT2) vs median 3' aQTL effect sizes taken across all tissues of the GTEx v8 atlas. Only lead SNPs with support in more than 1 tissue are included. **F** Predicted isoform log odds ratio of each model vs estimated aQTL effect sizes of the data from Mittleman et al. (2020) ($n = 58$). **G** Correlation between APARENT2 isoform log odds ratios and estimated eQTL effect sizes for $1,007$ intronic GTEx eQTLs and $2,225$ 3' UTR eQTLs, as a function of wildtype PAS usage as measured in tissue-pooled data from PolyADB v3. **H** Measured difference in isoform log odds between testis and tissue-pooled data from Lianoglou et al. (2013). The left distribution is the subset of proximal PASs with APARENT2 scores <0 and distal APARENT2 scores (in the same gene) >0. Inversely, the right distribution is the subset of PASs with proximal APARENT2 scores >0 and distal scores <0. The p-value is computed with a two-sided t-test. **I** Predicted vs measured tissue-specific difference in isoform proportion between HEK293 and the target tissue, on a held-out test set of $n = 500$ genes. Results are shown for either the most proximal, or next-to-last, PAS of each gene. **J** $+/-$ Spearman r correlation w.r.t baseline APARENT2 predictions of GTEx aQTL effect sizes, separated by GTEx tissue type, as a function of regression coefficient γ (see Methods) for each tissue-specific model. **K** Correlation of 3' aQTL effect sizes between tissues (GTEx v7). **L** Correlation (spearman r) of PAS usage between tissues in data from Lianoglou et al. (2013) ($n = 6,440$ genes). **M** Reconstructive interpretation mask for a GTEx SNP (rs4862524) with a weak tissue-specific effect in Testis. Boxplot shows differential PAS usage (difference in isoform log odds). The p-value is computed with a two-sided t-test.

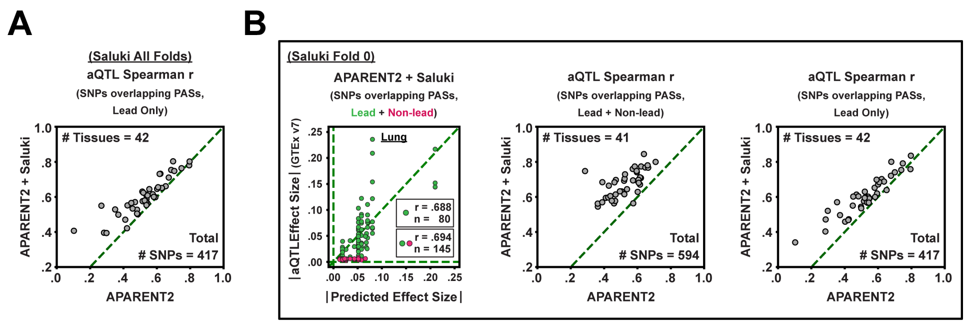

Figure S6: **A** Predicted vs measured 3' aQTL effect size spearman r's for lead SNPs only (GTEx v7). Each dot represents the spearman r in a given tissue. **B** Replicate analysis of results presented in main Fig. 6B-C, but using only the first fold of the Saluki model in combination with APARENT2 (in the main figure, the average prediction of 50 folds were used). Left: Absolute value of predicted vs measured aQTL effect sizes for lead SNPs and a matched set of non-lead SNPs overlapping PASs in Lung (GTEx v7). Final predictions are made by isotonic regression trained on all non-Lung SNPs. Middle: Predicted vs measured 3' aQTL effect size spearman r's per tissue for lead SNPs and a matched set of non-lead SNPs (GTEx v7). Right: Effect size spearman r's for lead SNPs only.

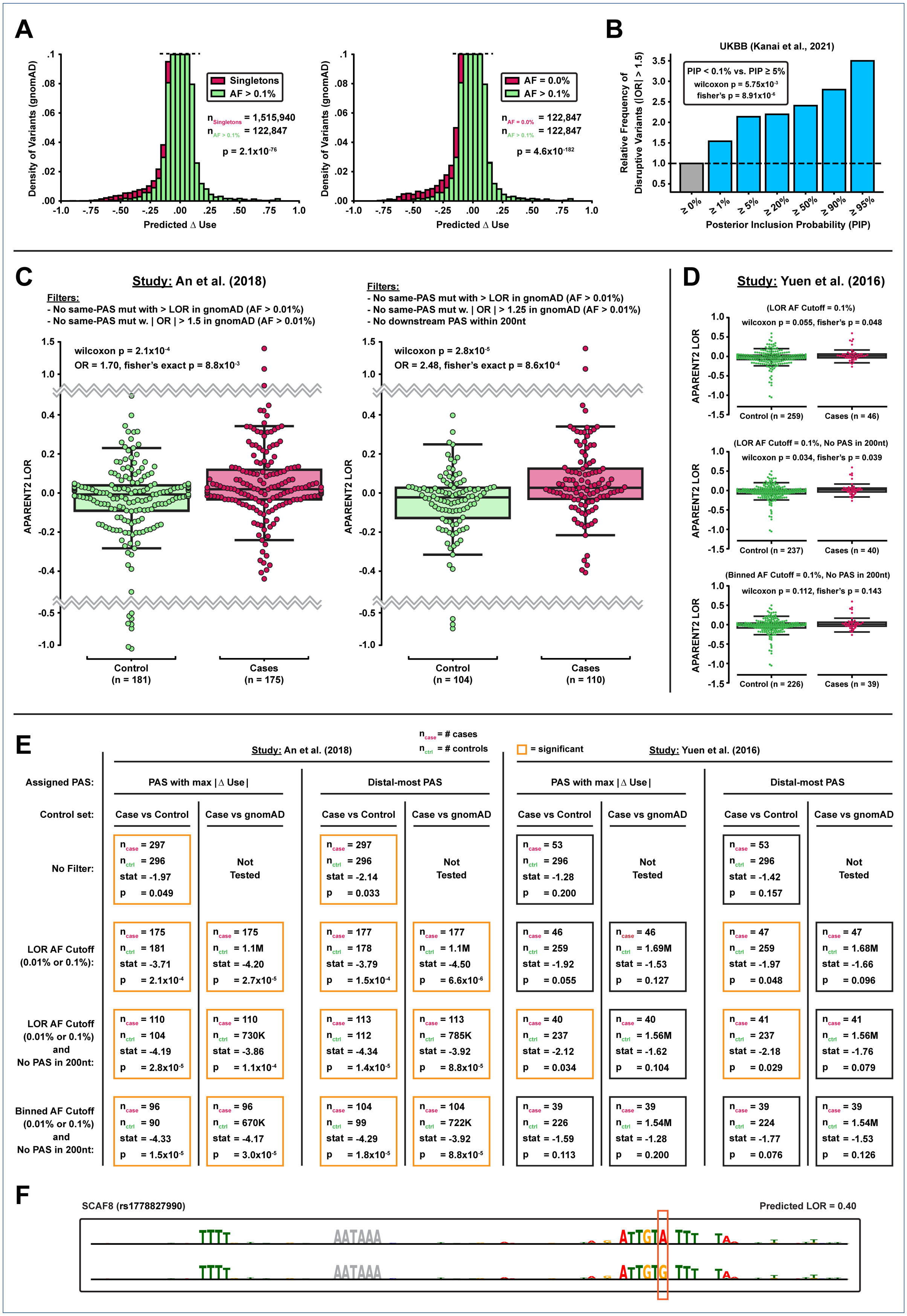
A
Density of Variants (gnomAD)
Predicted Δ Use
Singletons
AF > 0.1%
n Singletons = 1,515,940
n AF > 0.1% = 122,847
p = 2.1x10-76
AF = 0.0%
AF > 0.1%
n AF = 0.0% = 122,847
n AF > 0.1% = 122,847
p = 4.6x10-182
B
UKBB (Kanai et al., 2021)
PIP < 0.1% vs. PIP ≥ 5%
wilcoxon p = 5.75x10-3
fisher's p = 8.91x10-6
Relative Frequency of Disruptive Variants (|OR| > 1.5)
≥ 0% ≥ 1% ≥ 5% ≥ 20% ≥ 50% ≥ 90% ≥ 95%
Posterior Inclusion Probability (PIP)
C
Study: An et al. (2018)
Filters:
- No same-PAS mut with > LOR in gnomAD (AF > 0.01%)
- No same-PAS mut w. | OR | > 1.5 in gnomAD (AF > 0.01%)
wilcoxon p = 2.1x10-4
OR = 1.70, fisher's exact p = 8.8x10-3
APARENT2 LOR
Control (n = 181)
Cases (n = 175)
Filters:
- No same-PAS mut with > LOR in gnomAD (AF > 0.01%)
- No same-PAS mut w. | OR | > 1.25 in gnomAD (AF > 0.01%)
- No downstream PAS within 200nt
wilcoxon p = 2.8x10-5
OR = 2.48, fisher's exact p = 8.6x10-4
Control (n = 104)
Cases (n = 110)
D
Study: Yuen et al. (2016)
(LOR AF Cutoff = 0.1%)
wilcoxon p = 0.055, fisher's p = 0.048
Control (n = 259) Cases (n = 46)
(LOR AF Cutoff = 0.1%, No PAS in 200nt)
wilcoxon p = 0.034, fisher's p = 0.039
Control (n = 237) Cases (n = 40)
(Binned AF Cutoff = 0.1%, No PAS in 200nt)
wilcoxon p = 0.112, fisher's p = 0.143
Control (n = 226) Cases (n = 39)
E
n case = # cases
n ctrl = # controls
= significant
Study: An et al. (2018)
Study: Yuen et al. (2016)
Assigned PAS: PAS with max |Δ Use| / Distal-most PAS
Control set: Case vs Control / Case vs gnomAD
No Filter:
An, max: Case vs Control: n case = 297, n ctrl = 296, stat = -1.97, p = 0.049; Case vs gnomAD: Not Tested
An, distal: Case vs Control: n case = 297, n ctrl = 296, stat = -2.14, p = 0.033; Case vs gnomAD: Not Tested
Yuen, max: Case vs Control: n case = 53, n ctrl = 296, stat = -1.28, p = 0.200; Case vs gnomAD: Not Tested
Yuen, distal: Case vs Control: n case = 53, n ctrl = 296, stat = -1.42, p = 0.157; Case vs gnomAD: Not Tested
LOR AF Cutoff (0.01% or 0.1%):
An, max: n case = 175, n ctrl = 181, stat = -3.71, p = 2.1x10-4; n case = 175, n ctrl = 1.1M, stat = -4.20, p = 2.7x10-5
An, distal: n case = 177, n ctrl = 178, stat = -3.79, p = 1.5x10-4; n case = 177, n ctrl = 1.1M, stat = -4.50, p = 6.6x10-6
Yuen, max: n case = 46, n ctrl = 259, stat = -1.92, p = 0.055; n case = 46, n ctrl = 1.69M, stat = -1.53, p = 0.127
Yuen, distal: n case = 47, n ctrl = 259, stat = -1.97, p = 0.048; n case = 47, n ctrl = 1.68M, stat = -1.66, p = 0.096
LOR AF Cutoff (0.01% or 0.1%) and No PAS in 200nt:
An, max: n case = 110, n ctrl = 104, stat = -4.19, p = 2.8x10-5; n case = 110, n ctrl = 730K, stat = -3.86, p = 1.1x10-4
An, distal: n case = 113, n ctrl = 112, stat = -4.34, p = 1.4x10-5; n case = 113, n ctrl = 785K, stat = -3.92, p = 8.8x10-5
Yuen, max: n case = 40, n ctrl = 237, stat = -2.12, p = 0.034; n case = 40, n ctrl = 1.56M, stat = -1.62, p = 0.104
Yuen, distal: n case = 41, n ctrl = 237, stat = -2.18, p = 0.029; n case = 41, n ctrl = 1.56M, stat = -1.76, p = 0.079
Binned AF Cutoff (0.01% or 0.1%) and No PAS in 200nt:
An, max: n case = 96, n ctrl = 90, stat = -4.33, p = 1.5x10-5; n case = 96, n ctrl = 670K, stat = -4.17, p = 3.0x10-5
An, distal: n case = 104, n ctrl = 99, stat = -4.29, p = 1.8x10-5; n case = 104, n ctrl = 722K, stat = -3.92, p = 8.8x10-5
Yuen, max: n case = 39, n ctrl = 226, stat = -1.59, p = 0.113; n case = 39, n ctrl = 1.54M, stat = -1.28, p = 0.200
Yuen, distal: n case = 39, n ctrl = 224, stat = -1.77, p = 0.076; n case = 39, n ctrl = 1.54M, stat = -1.53, p = 0.126
F
SCAF8 (rs1778827990)
Predicted LOR = 0.40
AATAAA

Figure S7: **A** Left: Predicted Δ isoform proportions for singletons ($n = 1,515,940$) and common variants (AF >0.1%; $n = 122,847$) from gnomAD v3 that overlap annotated PASs in PolyADB V3. Right: Comparison of predictions for a matched set of unobserved PAS variants (AF = 0.0%; $n = 122,847$) and common variants (AF >0.1%) from gnomAD. **B** Relative frequency (enrichment) of fine-mapped SNPs from UK Biobank that overlap annotated PASs ($n = 90,356$) with a predicted absolute-valued isoform odds ratio >1.5, as a function of more stringent posterior inclusion probabilities (PIP). **C** Distribution of predicted isoform log odds ratios among ASD cases and controls from the WGS study of An et al. (2018). Left: Case- and control variants are removed if they occur in PASs that have common variants in gnomAD (AF >0.01%) with larger effect size (log odds ratio) than the investigated variant or common variants that have an absolute odds ratio larger than 1.5 ($n_{\text{control}} = 181$, $n_{\text{cases}} = 175$). Right: Additional removal of variants that occur in PASs with a downstream PAS within 200nt in PolyADB V3 ($n_{\text{control}} = 104$, $n_{\text{cases}} = 110$). A more stringent odds ratio threshold of 1.25 was used. This is the same filtering procedure as in main Fig. 7G, but here the allele count of variants within the same PAS with similar effect sizes have not been aggregated prior to filtering against gnomAD (see Methods for details on the variant filtering procedure). **D** Replicate analysis where the case variants are from the WGS study of 200 families from Yuen et al. (2016) (the control variants come from An et al., 2018). The three filtering steps used in supplementary Fig. S7C and main Fig. 7G are also used here: (1) filtering variants with neighboring common mutations in gnomAD (AF >0.1%; $n_{\text{control}} = 259$, $n_{\text{cases}} = 46$), (2) additionally removing variants with downstream protective PASs within 200nt ($n_{\text{control}} = 237$, $n_{\text{cases}} = 40$), and (3) same filtering criteria as (2) but gnomAD AFs are re-calculated by aggregating allele counts of similar predicted effect size in the same PAS ($n_{\text{control}} = 226$, $n_{\text{cases}} = 39$). **E** Summary of statistical tests performed on the cohort data from An et al. (2018) (left table) and Yuen et al. (2016) (right table). P-values were calculated using Wilcoxon rank-sum tests. The rows denote different filtering criteria and the columns denote the control group used (controls either come from An et al. or from an identically filtered view of gnomAD). Two different methods were tested for assigning variants to PASs (when variants overlap two nearby PASs): (1) the PAS resulting in largest predicted variant effect size or (2) the distal-most PAS. Significant tests are marked with an orange border. **F** Mask-based interpretation of an ASD-associated PAS mutation rs1778827990 in SCAF8.

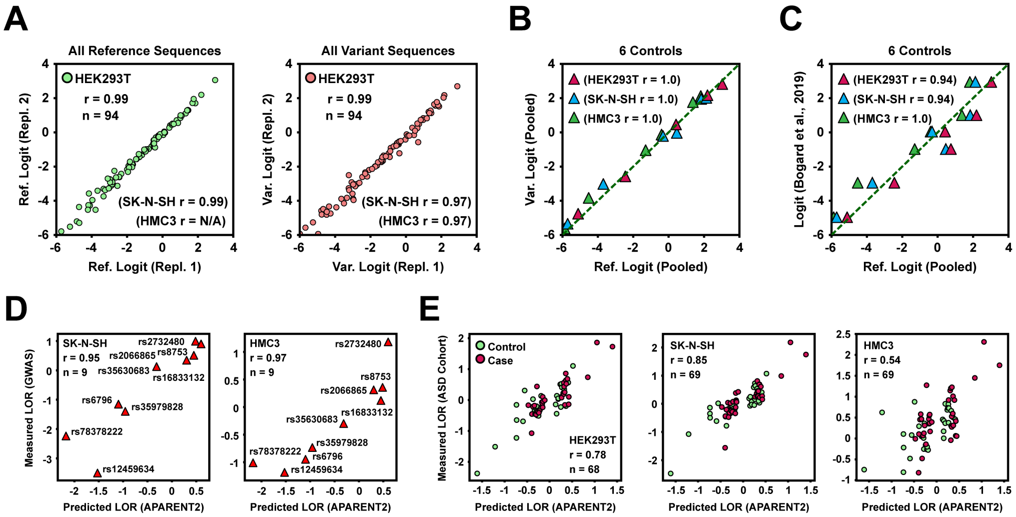

Figure S8: **A** Replicate correlation of proximal isoform logits as measured in the plasmid reporter MPRA for the subset of the 100 assayed PASs that had a minimum of 5 supporting reads. Left: Measured logits of reference (wildtype) PASs. Right: Measured logits of variant (alternate) PASs. The scatter plots show the results for HEK293T, while summary correlation metrics for SK-N-SH and HMC3 are annotated in the plots. HMC3 had only one biological replicate with sufficient read depth for the reference library. **B** Proximal isoform logits of 6 control PASs that were assayed (without any designed mutations) in both the reference and variant libraries. The isoform logits were estimated by pooling the counts across replicates. **C** Correlation between the 6 control PASs of the reference library and their estimates from the MPRA of Bogard et al. (2019) where they had previously been measured. **D** Predicted vs measured variant effect sizes (log odds ratios, *LORs*) of 9 assayed GWAS SNPs. Results are shown for SK-N-SH and HMC3. **E** Predicted vs measured variant LORs of the subset of assayed Autism variants with a minimum of 50 reads (pooled replicates). Green = control variants, Magenta = case variants.

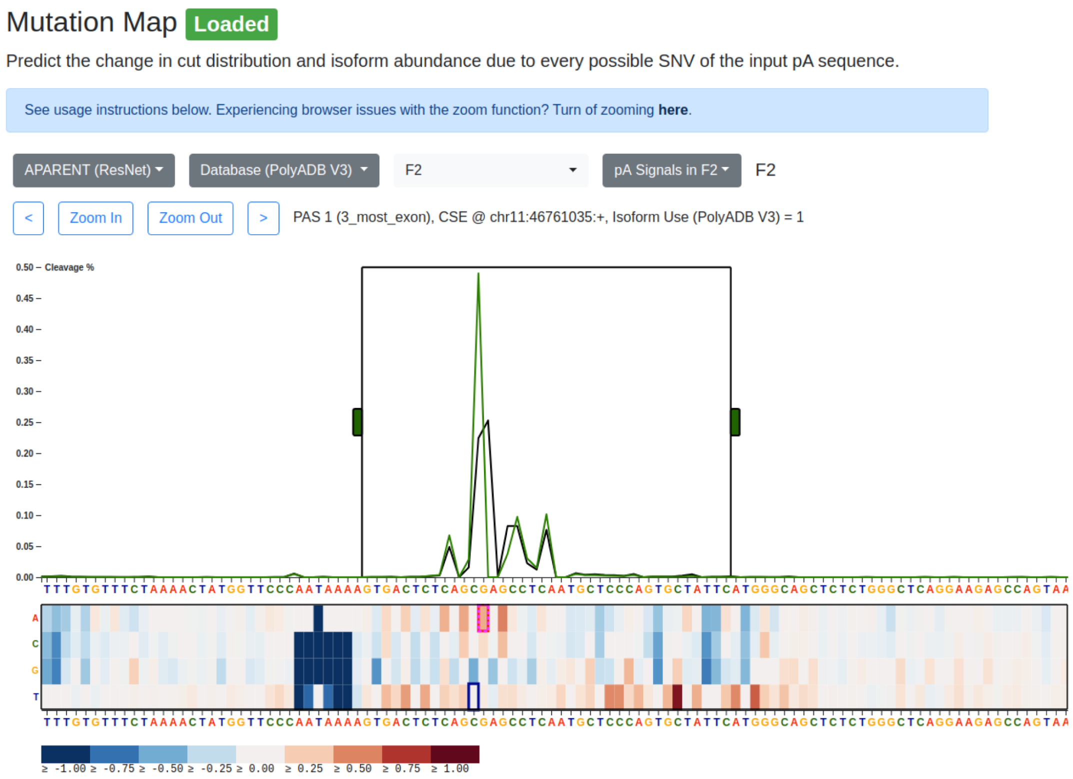

Figure S9: Screenshot of the polyadenylation variant prediction web tool. The tool allows users to perform in-silico saturation mutagenesis and interactively investigate the altered cleavage distribution due to every individual variant.
