# Supplementary material for: Deciphering the impact of genetic variation on human polyadenylation using APARENT2
Source: Genome Biol. 2022 Nov 5;23:232. doi: 10.1186/s13059-022-02799-4 (PMC9636789; doi:10.1186/s13059-022-02799-4)
Supplement: Supplementary file 3 — Additional file 3. Review history. [file 13059_2022_2799_MOESM3_ESM.docx]

Review History

**First round of review**

**Reviewer 1**

**Are you able to assess all statistics in the manuscript, including the appropriateness of statistical tests used?** Yes, and I have assessed the statistics in my report.

**Comments to author:**

In this paper the authors extend their previous work on DL models of PA and APA. The new model is considerably deeper and uses residual blocks, as well as a novel loss function reminiscent of their BPNet work, with a base pair resolution term and a global term. On the same (although reprocessed) data, the new model "APARENT2" outperforms both the original model and other available models on a variety of tasks including held out prediction on the MPRA and predicting GTEx and HapMap YRI APA QTLs. The authors explore model interpretation using their scrambling/mask approach and do a nice job dissecting some specific examples. They also show depletion of predicted APA disrupting common variants, and an enrichment in de novo mutations in ASD.

Overall this is a very comprehensive study and I recommend it for publication. My one small criticism is that in some of the QTL/GWAS analyses it would have been preferable to use fine-mapped variants rather than lead SNPs, but this is a considerable undertaking and would probably not substantially change the results (although it might make them look even better!)

Minor comment:

"For example, when we intersected our transcriptome-wide PAS variant predictions against the GWAS catalog (n = 1,233) we observed common SNPs with both large (>1.5-fold) and small (<1.5-fold) predicted effect sizes that are strongly associated with phenotypes such as elevated cholesterol and Attention- deficit / Hyperactivity Disorder."
These aren't fine-mapped so I don't think you can conclude much from this or that it adds much to the argument: the variability in effect sizes might be due to different degrees of tagging the actual causal variant.

**Reviewer 2**

**Are you able to assess all statistics in the manuscript, including the appropriateness of statistical tests used?** Yes, and I have assessed the statistics in my report.

**Comments to author:**

The paper represents the next installment of the machine learning method named APARENT for infer 3'-cleavage and polyadenylation from DNA sequence. The main difference between APARENT and APARENT2 is the use of a deep residual block architecture. The authors performed extensive evaluation of APARENT2 on such datasets as MRPA, HGMD, HapMap Yoruba human cell lines, and so on. The method showed capability to correctly predict  3'-cleavage and polyadenylation from DNA sequence. Linder et al performed extensive comparison with existing methods and showed that APARENT2 outperformed or had similar performance with the rest of the methods. The authors also showed several good examples how the proposed neural network can be used for sequence interpretation. Linder et al. updated web-server and made the easy usage of APARENT2 possible. I would like to note that the preparation all datasets for further analysis is well-documented. Overall, it is a good paper that contains interesting results. There are several typos and a minor clarification point, which the authors should address.

Minor points / typos:
1. The extra consistency term in the loss function should be discussed. Why is it needed?
2. There are two identical discussion section in the paper. One of them should be removed.
3. Figure 3E: APARENT-ResNet -> APARENT2?
4. Page 22, line 14-15, - extra bracket.

**Authors Response**

**Point-by-point responses to the reviewers’ comments:**

***We thank the reviewers for their helpful comments. We have provided detailed responses to reviewer reports further below where we address their specific concerns and suggestions.*

*In addition to the changes made due to feedback from the reviewers, we have also made a number of improvements to the manuscript after feedback from colleagues and peers. Specifically, the revised manuscript includes the following updated or new analyses:*

*1. Improved use of half-life predictions: On Page 4, Line 24 of the updated manuscript (Section “A Residual Neural Network for Predicting…”), we now use the last hidden-layer outputs of the Saluki model as half-life covariates to our APA model instead of the final half-life predictions, and get consistently better cross-validation performance on held-out data. The presumed reason is that APARENT2 knows about stability downstream, but not upstream, of the CSE and thus the hidden-layer outputs of Saluki can correct APARENT2 better than the final half-life scores. These new results are reflected in Figure 1F-G.

2. Split variant prediction and interpretation into two figures: The original Figure 2 included both MPRA benchmark results and variant interpretations. In the updated manuscript, the section “Predicting the Impact of Variants on Polyadenylation…”, starting on Page 4, Line 53 (and Figure 2) focuses solely on the MPRA benchmarks. The new section “A Catalog of Higher-Order Polyadenylation…”, starting on Page 5, Line 32 (Figure 3) is dedicated to interpretation. We feel that a dedicated figure on interpretability is in line with the theme of the GB special issue. Note that this is mainly a structural change, and the content is unaltered except for additional interpretation examples.

3. Reordered some of the content: The section “Silent Hexamer Mutations are Protected…” of the original manuscript used to come after the QTL analyses. In the updated manuscript, we have moved the section up and it now starts on Page 6, Line 28, immediately after the MPRA benchmarks and interpretation. This makes more logical sense since this analysis deals with MPRA data too.

4. We have collected new experimental MPRA data in multiple cell lines (including microglia-derived HMC3 cells), in order to validate the APARENT2 predictions for a subset of Autism variants as well as a few fine-mapped GWAS SNPs. These results, which we feel strengthen the paper considerably, are presented in the new section “An MPRA of Clinically Relevant Variants…” on Page 10, Line 57 (and new Figure 8).

5. Based on feedback from peers, we have made minor improvements or clarifications to the text throughout the paper (mainly correcting grammatical errors or providing more details).***


--- Reviewer #1: ---

In this paper the authors extend their previous work on DL models of PA and APA. The new model is considerably deeper and uses residual blocks, as well as a novel loss function reminiscent of their BPNet work, with a base pair resolution term and a global term. On the same (although reprocessed) data, the new model "APARENT2" outperforms both the original model and other available models on a variety of tasks including held out prediction on the MPRA and predicting GTEx and HapMap YRI APA QTLs. The authors explore model interpretation using their scrambling/mask approach and do a nice job dissecting some specific examples. They also show depletion of predicted APA disrupting common variants, and an enrichment in de novo mutations in ASD.

Overall this is a very comprehensive study and I recommend it for publication. My one small criticism is that in some of the QTL/GWAS analyses it would have been preferable to use fine-mapped variants rather than lead SNPs, but this is a considerable undertaking and would probably not substantially change the results (although it might make them look even better!)

***Response: We thank the reviewer for their helpful comments and suggestions. We agree that the GWAS analysis would improve if we used fine-mapped variants. Thus, in the updated manuscript, on Page 9, Line 48 (and associated Figure 7D) we have replaced the previous analysis with fine-mapped GWAS variants from two sources (the credible set of CAUSALdb and all SNPs from a UK Biobank fine-mapping study). In Supplementary Figure S7B, we show that higher-impact predictions are enriched in UKBB variants with increasingly larger PIP. In Figure 7D, we co-localize our predictions with many fine-mapped variants from CAUSALdb with PIP > 0.5.*

*We do think that the use of lead SNPs is less of a problem for the QTL analysis; while the potential problem of LD persists, it is not as severe as GWAS since the QTLs are called from WGS data rather than Tag SNPs (which are optimized for high LD). Since it would be considerable work to fine-map the aQTLs (and we don’t expect this to substantially change the relative benchmark results), we kept the use of lead SNPs. However, to provide further evidence that lead aQTLs contain useful signal, we have included a new short section starting on Page 8, Line 45 (with associated new Figure 6) where we use both APARENT2 and Saluki (half-life predictor) to improve the correlation on aQTL effect sizes. In Figure 6B, we see a clear separation between lead SNPs and matched non-lead SNPs.

We have also included a replicate aQTL benchmark for the newer GTEx v8 catalog in Supplementary Figure S5C-D, which shows that the results in Main Figure 5 reproduce even with a larger coverage of variants found in the WGS + RNA-seq data. Put together, we hope and believe we have convinced the reviewer that the aQTL prediction results are sound.***

Minor comment:

"For example, when we intersected our transcriptome-wide PAS variant predictions against the GWAS catalog (n = 1,233) we observed common SNPs with both large (>1.5-fold) and small (<1.5-fold) predicted effect sizes that are strongly associated with phenotypes such as elevated cholesterol and Attention- deficit / Hyperactivity Disorder."
These aren't fine-mapped so I don't think you can conclude much from this or that it adds much to the argument: the variability in effect sizes might be due to different degrees of tagging the actual causal variant.

***Response: This sentence has since been removed from the updated manuscript since we have replaced the previous GWAS analysis with new results based on fine-mapping studies.***


--- Reviewer #2: ---

The paper represents the next installment of the machine learning method named APARENT for infer 3'-cleavage and polyadenylation from DNA sequence. The main difference between APARENT and APARENT2 is the use of a deep residual block architecture. The authors performed extensive evaluation of APARENT2 on such datasets as MRPA, HGMD, HapMap Yoruba human cell lines, and so on. The method showed capability to correctly predict 3'-cleavage and polyadenylation from DNA sequence. Linder et al performed extensive comparison with existing methods and showed that APARENT2 outperformed or had similar performance with the rest of the methods. The authors also showed several good examples how the proposed neural network can be used for sequence interpretation. Linder et al. updated web-server and made the easy usage of APARENT2 possible. I would like to note that the preparation all datasets for further analysis is well-documented. Overall, it is a good paper that contains
interesting results. There are several typos and a minor clarification point, which the authors should address.

***Response: We thank the reviewer for their comments and provide responses to the points raised below.***

Minor points / typos:
1. The extra consistency term in the loss function should be discussed. Why is it needed?

***Response: We have added the following motivation for the use of the consistency term in the Methods section, on Page 14, Line 34:

“We found empirically that the extra consistency term produced better isoform fold-change predictions for downstream variant prediction tasks, presumably by increasing the importance of predicting cleavage accurately within the region specified by this loss term (which is where the majority of proximal cleavage occurs for most PASs).”

In other words, the consistency term is a way of up-weighting the cost of predicting cleavage incorrectly in the most important region of the polyadenylation signal (namely downstream of the CSE), at the implicit expense of down-weighting the loss at other positions.***

2. There are two identical discussion section in the paper. One of them should be removed.

***Response: We thank the reviewer for pointing this out; we have now removed the duplicate section.***

3. Figure 3E: APARENT-ResNet -> APARENT2?

***Response: We have updated the figure with the corrected name (APARENT2).***

4. Page 22, line 14-15, - extra bracket.

***Response: We have removed the extra bracket.***
